# Virus Diseases of Cereal and Oilseed Crops in Australia: Current Position and Future Challenges

**DOI:** 10.3390/v13102051

**Published:** 2021-10-12

**Authors:** Roger A. C. Jones, Murray Sharman, Piotr Trębicki, Solomon Maina, Benjamin S. Congdon

**Affiliations:** 1 UWA Institute of Agriculture, University of Western Australia, Crawley, WA 6009, Australia; 2 Queensland Department of Agriculture and Fisheries, Ecosciences Precinct, P.O. Box 267, Brisbane, QLD 4001, Australia; murray.sharman@qld.gov.au; 3 Grains Innovation Park, Agriculture Victoria, Department of Jobs, Precincts and Regions, Horsham, VIC 3400, Australia; piotr.trebicki@agriculture.vic.gov.au (P.T.); solomon.maina@agriculture.vic.gov.au (S.M.); 4 Department of Primary Industries and Regional Development, South Perth, WA 6151, Australia; Benjamin.Congdon@dpird.wa.gov.au

**Keywords:** virus diseases, Australia, cereals, oilseeds, epidemiology, management, losses, history, research priorities, future challenges

## Abstract

This review summarizes research on virus diseases of cereals and oilseeds in Australia since the 1950s. All viruses known to infect the diverse range of cereal and oilseed crops grown in the continent’s temperate, Mediterranean, subtropical and tropical cropping regions are included. Viruses that occur commonly and have potential to cause the greatest seed yield and quality losses are described in detail, focusing on their biology, epidemiology and management. These are: barley yellow dwarf virus, cereal yellow dwarf virus and wheat streak mosaic virus in wheat, barley, oats, triticale and rye; Johnsongrass mosaic virus in sorghum, maize, sweet corn and pearl millet; turnip yellows virus and turnip mosaic virus in canola and Indian mustard; tobacco streak virus in sunflower; and cotton bunchy top virus in cotton. The currently less important viruses covered number nine infecting nine cereal crops and 14 infecting eight oilseed crops (none recorded for rice or linseed). Brief background information on the scope of the Australian cereal and oilseed industries, virus epidemiology and management and yield loss quantification is provided. Major future threats to managing virus diseases effectively include damaging viruses and virus vector species spreading from elsewhere, the increasing spectrum of insecticide resistance in insect and mite vectors, resistance-breaking virus strains, changes in epidemiology, virus and vectors impacts arising from climate instability and extreme weather events, and insufficient industry awareness of virus diseases. The pressing need for more resources to focus on addressing these threats is emphasized and recommendations over future research priorities provided.

## 1. Introduction

On a global scale, virus disease outbreaks, epidemics and pandemics threaten all kinds of crops and, depending upon individual circumstances, cause damage varying from total crop failure to small-scale losses. They threaten crop plants not only by impairing their growth and vigor thereby diminishing gross yields, but also by damaging produce quality thus decreasing marketable yields. Agricultural globalization, climate change-driven influences and other factors, such as pesticide resistance in virus vectors, are aggravating their spread and making their control more difficult [1,2,3,4,5,6,7,8,9,10,11]. In 2014, the annual global economic impact of crop virus diseases was estimated at more than US$30 billion [12] but has increased since then. Australian agriculture is threatened by many of the same virus diseases that afflict crops in other parts of the world [13], and viruses introduced by growing infected introduced crops threaten Australia’s unique native flora [14]. Fortunately, however, a strict biosecurity system is in place to help minimize the likelihood that others will arrive and become established [15].

Native plants were used extensively as crops by Australia’s Aboriginal population before European settlement commenced in 1788 [16,17]. However, today, with very few exceptions, the continent’s agriculture consists entirely of crops domesticated on other continents [18]. As it has a diverse range of climatic zones, from temperate to tropical, and varies in altitude from sea level to mountains reaching up to 2228 m, Australia is able to produce the full spectrum of temperate to tropical crops grown globally. Agriculture accounts for 55% of Australia’s land use (427 million hectares, excluding timber) and 11% of its exports, with 70% of its agricultural production is exported [19]. The principal agricultural crops are the cereals–wheat (*Triticum aestivum*), barley (*Hordeum sativum*), oats (*Avena sativa*), triticale (*Triticum* genus × *Secale cereale*), sorghum (*Sorghum bicolor*), maize and sweet corn (*Zea mays*), and rice (*Oryza sativa*); oilseeds–canola (=oilseed rape; *Brassica napus*), sunflower (*Helianthus annuus*), soybean (*Glycine max*), peanut (*Arachis hypogea*) and cottonseed (*Gossypium hirsutum*); and grain legumes–lupin (*Lupinus* spp.), chickpea (*Cicer arietinum*), field pea (*Pisum sativum*), faba bean (*Vicia faba*), lentil (*Lens culinaris*) and mungbean (*Vigna radiata*). In addition, Australia grows sugarcane (*Saccharum officinarum*) and a wide range of horticultural crops (i.e., fruits and vegetables), relies on improved pastures to provide the main the feed-base for its extensive wool, dairy and meat industries, and has a large timber industry. The most important agricultural crop is wheat with c. 20 million tonnes produced annually. Barley, canola, cottonseed and rice are also produced on a very large-scale, with production of other cereal and oilseed crops, and of grain legumes occurring on a smaller scale [19]. These crops are threatened by a wide range of virus diseases which diminish both gross and marketable yields. To address this challenge, a considerable amount of research has been undertaken since the 1950s aimed at obtaining a better understanding of their biology and epidemiology, and how to manage them.

The book ‘*Viruses of Plants in Australia*’ constitutes an invaluable resource that briefly summarized all studies on plant viruses of cultivated and wild plants undertaken up to 1987 in Australia [13]. Several past general Australian plant disease reviews covered pasture and forage legumes and/or grasses. Each of these included a summary of the virus disease situation in these species up to the time they were published [20,21,22,23,24]. Two other past general plant disease reviews did this with cool season grain legume and mustard (*B. juncea* and *B. carinata*) crops [25,26]. However, very few past reviews focused solely upon virus diseases in Australia. Those that have done this were limited to virus diseases of pasture and forage grasses and legumes [27,28,29,30,31,32] and the grain legume crop lupin [33,34], or to yellow dwarf diseases (YDD) in wheat and Australian ‘grasslands’ [35]. However, none focused exclusively on virus diseases of cereals or oilseeds in Australia. Our review aims to rectify this situation by providing an up-to-date, comprehensive review covering all past and recent Australian research undertaken on viral diseases of these two types of crop across the full spectrum of the continent’s agroecological zones. It starts by providing brief general background information concerning the scope of the Australian Grains Industry, plant virus epidemiology, the general principles of virus disease management and yield loss estimation, and listing all viruses recorded infecting Australian cereal and oilseed crops. Next, it provides more detailed accounts of Australian research on virus diseases of these two crop categories, makes recommendations for future research, and describes how new virus and vector incursions from overseas, insecticide resistance in virus vectors and climate change are magnifying their epidemics and the damage they cause.

## 2. Background Information

### 2.1. Australian Cereals and Oilseeds Industries

**Figure 1:**
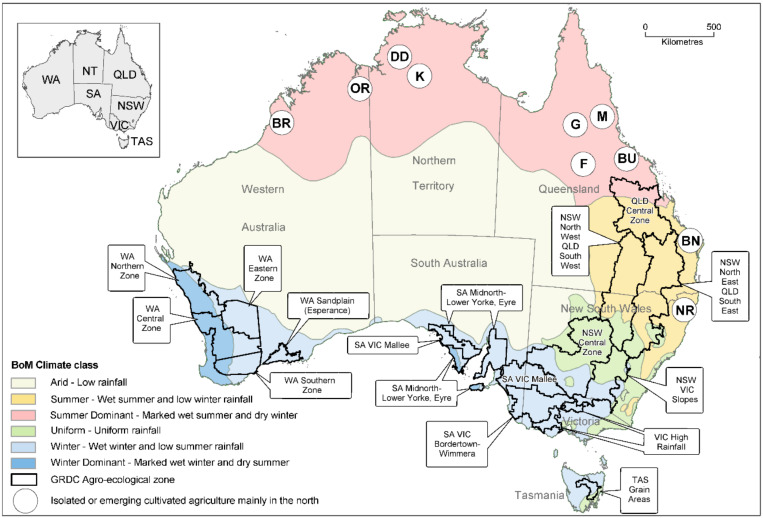
Map of the Australian continent. The state and territory acronyms shown are NSW (New South Wales), NT (Northern Territory), QLD (Queensland), SA (South Australia), TAS (Tasmania), VIC (Victoria) and WA (Western Australia). The map shows: (i) the principal regions where grain crops are grown differentiated by Australian Bureau of Meteorology (BOM) climate classes, and (ii) smaller isolated regions of irrigated grain production designated by letters: Broome (BR) and the Ord River Irrigation Area (OR) in WA; Douglas/Daly (DD) and Katherine (K) regions in the NT; Burdekin (BU), Bundaberg (BN), Gilbert (G), Flinders (F), Mareeba (including Atherton and Ravenshoe) and (M) regions in QLD; and Northern Rivers (NR) region in NSW. The agro-ecological zones bounded black lines constitute a modified version of those delineated by the Australian Grains Research and Development Corporation (GRDC) [51]. Image credit @Department of Primary Industries and Regional Development/P. Goulding.

In Australia’s grainbelt regions, agriculture occurs on a vast scale, with individual fields up to, and sometimes exceeding, 250 hectares in size. About 22 million hectares are planted with crops annually, and this cropping depends on large machines with minimal labor and chemical inputs [18,19,36,37]. The principal grain producing region extends from central Queensland (QLD) south westwards through New South Wales (NSW) to Victoria (VIC), and then north eastwards to South Australia (SA) (Figure 1). It also includes much of south-west Western Australia (WA), although the intervening semi-arid Nullarbor Plain region is unsuitable for agriculture. Cropping in Australian grainbelts is mostly rainfed. In the south-west and southern grainbelt regions rainfall is winter dominant (wet winter, dry summer), but in southern and central NSW rainfall occurs all-year-round. In Northern NSW, and in south and central QLD, the summer is wet and winter rainfall is low. The climate is classified as subtropical in central and south-eastern QLD and northern NSW, Mediterranean in south-west WA and southern SA, and temperate in VIC and southern NSW, and in the small grain-producing region in the island state of Tasmania (TAS) [19,38,39,40]. The amount of rainfall received decreases with distance from the coast, and three cropping zones are present which differ in the rainfall each receives annually, high (700–450 mm), medium (450–225 mm) or low (225–175). High rainfall zones produce the greatest yields, whereas low rainfall zones produce the lowest yields and are more vulnerable to drought stress conditions and crop failure [41,42,43]. In the southern grainbelts (WA, SA, VIC, TAS, and both southern and central south NSW), the rain-fed crops grown over winter are wheat, barley, oats, triticale, canola, safflower (*Carthamus tinctorius*), field pea, faba bean, lupin, chickpea and lentil. However, there are regional differences, e.g., safflower and chickpea being absent in TAS, safflower in south-west WA, and lupin being a much bigger crop in WA than elsewhere [44]. In the northern grainbelt (central north and north NSW, south and Central QLD), the rain-fed crops grown over winter are wheat, barley, oats, triticale, canola, safflower, linseed, field peas, lupins, chickpea and lentils, whereas those grown over summer are maize, sweet corn, sorghum, sunflower, mungbean, soybean, peanut and cotton. Again, there are regional differences, e.g., in eastern Australia no triticale, safflower, linseed (*Linum usitatissimum*), lupin, field pea or faba bean is grown in central QLD, and lupin is only grown in central north NSW [19,44]. Rice production differs in that it depends on irrigation. Along with irrigated maize and sweet corn, rice is grown predominantly in the southern Murray–Darling Basin region of NSW [45,46,47]. In tropical northern Australia, there are pockets of irrigated grain and oilseed production in: (i) the Ord River Irrigation Area and Broome in the Kimberley region of WA; (ii) the Douglas/Daly and Katherine regions in the Northern Territory (NT); and (iii) the Burdekin, Gilbert, Flinders and Mareeba (including Atherton and Ravenshoe) regions in Queensland (QLD). The grain and oilseed crops grown in these locations include rice, cotton, sorghum, maize, sweet corn, pearl millet (*Pennisetum glaucum*), chickpea, soybean, cowpea, peanut, mungbean, sesame (*Sesamum indicum*), sunflower and safflower [48,49,50]. In addition, in subtropical south-eastern QLD and northern NSW, irrigated crops of peanut and soybean are grown in the Bundaberg and maize and sunflower in the Northern Rivers regions, respectively.

### 2.2. Epidemiology

**Figure 2:**
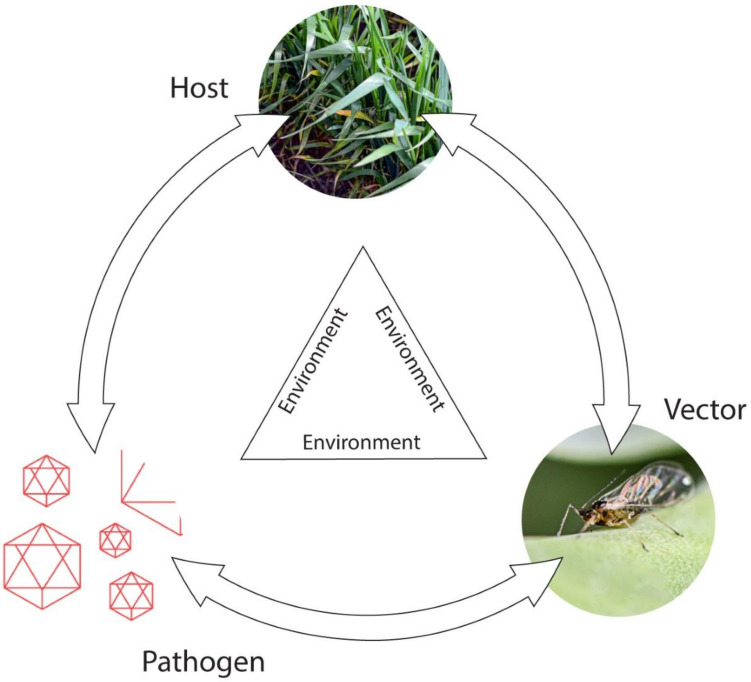
Disease triangle for vector-borne virus pathosystem scenarios. Arrows represent interactions between the different triangle components.

To manage plant virus diseases effectively, knowledge of their epidemiology is crucial, and a systems biology approach is needed to provide a holistic understanding of factors triggering their epidemics. Obtaining this knowledge necessitates studying each virus pathosystem in different agro-ecological situations. The disposition, size and characteristics of the initial source of virus infection, how the virus spreads from plant-to-plant and over distance to new locations, and how it survives outside the principal growing period, all constitute critical factors influencing epidemic development [52,53,54,55]. Epidemic magnitude often varies widely with locality and year due to the overriding influence of local weather conditions, especially temperature and rainfall, on vector populations. Moreover, which type of vector is involved, and, with arthropod-borne viruses, whether virus transmission is persistent or non-persistent, all strongly influence epidemic development. Additional factors determining the shape and magnitude of epidemic scenarios include whether (i) primary virus sources are internal or external; (ii) temporal spread patterns polycyclic or monocyclic; (iii) spatial patterns clumped or random; and (iv) vectors single or multispecies. Thus, many intrinsic and extrinsic factors influence the trilateral interactions between host plants, viruses and vectors (Figure 2). The diverse scenarios arising from these complex interactions dictate the spatiotemporal virus spread patterns that arise [52,53,54,55].

### 2.3. General Management Principles

The remarkable biological and genetic diversity of both viruses and vectors makes implementation ‘one-size-fits-all’ control solutions impossible. Also, when used on their own single control measures rarely reduce virus-induced yield losses adequately. Fortunately, however, integrated disease management approaches can deliver appropriate solutions that are not only robust but also economically, environmentally, and socially sustainable [4,33,53,56,57,58,59].

Approaches of this type must be both intelligent and adaptable, and include locally appropriate choices that avoid damage to local ecosystems. Their development depends on an understanding of: (i) the epidemiological factors that give rise to harmful virus epidemics and (ii) the effectiveness of each control measure being deployed. The main kinds of virus control measures are phytosanitary, cultural, chemical and host resistance. In integrated disease management, different kinds of control measures that vary in the ways they operate are combined so they effectively target each combination of virus and crop and situation (Table 1). Individual integrated disease management components consist of diverse phytosanitary and cultural measures which minimize the virus source or diminish its spread, virus-resistant cultivars which minimize virus spread, and selective pesticides that not only kill vectors but also act in a way that is environmentally responsible [33,53,56,57,58,59].

In addition, new technologies offer considerable promise toward more effective future plant virus disease management in the field. For example, thermal, multispectral, hyperspectral, and other forms of optical sensors are increasingly becoming effective at discriminating between virus-diseased and healthy plants, determining virus and virus vector occurrence, and helping to predict virus disease induced yield losses [60,61]. Integrating them with precision agriculture to enable the targeting of control measures where they have greatest impact holds great potential for the future in achieving effective pre-emptive virus disease control [32,53,61]. Moreover, speed breeding [62], and genetic modification using RNA silencing and CRISPR-Cas hold considerable promise for rapidly breeding virus-resistant crop cultivars [63]. Furthermore, exogenous delivery of dsRNA [64], nanotechnology [65] and biostimulants [66] applied post emergence have the potential to help reduce the spread and impact of virus diseases. However, for these technological innovations to be optimized, they need to be integrated with a good understanding of how epidemics develop, and the other factors involved, especially the cultural and climatic factors, and be adapted to each cropping situation [32,53,61].

### 2.4. Viruses Infecting Cereal and Oilseed Crops

In Australia, as in other world regions, in cereal and oilseed crops a wide diversity of virus diseases diminish gross yields and seed quality. These losses are greatest when widespread virus infection occurs at early stages of crop growth resulting in large-scale plant stunting or systemic necrosis. However, provided widespread early infection has taken place, significant crop losses may still occur when foliage symptoms are mild and stunting minor [53]. How important a virus is to the Australian grains industry depends on its overall incidence and the magnitude of the losses it causes in seed yield and quality losses, and the economic value of the crop commodity. Thus, some may cause mild symptoms and smaller losses yet still be important due to their widespread occurrence and the high economic value of the crop type, whereas others may cause severe losses but bedeemed of low overall importance because they are uncommon. When a virus reaches high incidences in a particular crop, the yield loss that arises depends greatly upon: (i) the sensitivity of the cultivar grown; (ii) the virus strain that is prevalent; (iii) the abundance of vectors; (vi) the presence of other biotic and abiotic stresses; (v) environmental conditions, and (vi) interactions between all of these factors. Table 2 lists the more important viruses found, the principal crops they infect, the maximum percentage yield losses recorded and their known effects on seed quality. It also shows the main types of virus symptoms, which insect or mite vectors transmit them, if they are seed-borne and their occurrence in different Australian grain growing regions occupying specific climatic zones. Table 3 provides the same information for currently less important viruses. In addition, Table 2 and Table 3 show that, currently, there are no viruses recorded infecting rice in Australia. However, two mastreviruses named rice latent viruses 1 and 2 were found in infecting wild rice species in the Cape York Peninsula in tropical northern QLD [67] so this likely reflects lack of attention paid by virologists and rice farmers rather than their complete absence. Similarly, there are no records of virus infection in linseed.

The book ‘Viruses of Plants in Australia’ published in 1988 [13] constitutes an extremely useful data resource covering early Australian studies on virus diseases of cereal and oilseed, and other crops. The same applies to historical collections of Australian virus isolates including from cereal and oilseed crops, especially those containing viruses from the pre-sequencing era [68]. Combining the wealth of historical information about viruses studied over more than four decades available in this book with new complete genomic sequence data about virus isolates studied during that era now obtainable from historical culture collections offers exciting prospects. These include avoiding new names being incorrectly allocated to previously named viruses, providing original reference virus sequences, and providing access to pre-sequencing era data on biological and other parameters. They also include resolution of other confounding virus naming issues, and identification of unknown viruses preserved many years ago.

### 2.5. Quantification of Yield Losses

The maximum yield loss data in Table 2 and Table 3 mostly represent worse-case scenario situations and may sometimes come from comparisons between virus-infected and healthy plants grown in pots in the glasshouse or as spaced plants or single row plots in the field. However, yield loss data from large plots growing in replicated field experiments in which virus infection is spreading between plants naturally provides the most meaningful yield loss data. This is because plants become infected at different growth stages and compensatory growth of healthy plants growing next to infected plants takes place. Greatest losses occur when most plants become infected at vulnerable early growth stages [56]. Replicated field experiments generally provide the best yield data when their plots become infected following exposure to an evenly distributed virus source and are separated from one another by wide non-host crop barriers that minimize plot-plot-spread. Use of non-host barriers is particularly important with non-persistently vector-borne viruses [56]. For the important virus host combinations in Table 2, there are examples of past Australian field experimentation that provided meaningful yield loss data for cereal and oilseed crops. These include ones where the infecting virus was barley yellow dwarf virus (BYDV; genus *Luteovirus*, family *Tombusviridae*) in wheat, barley or oats [69,70,71,72,73], wheat streak mosaic virus (WSMV; genus *Tritimovirus*, family *Potyviridae*) in wheat [74], Johnsongrass mosaic virus (JGMV; genus *Potyvirus*, family *Potyviridae*) in maize or sorghum [75,76]; and turnip yellows virus (TuYV; genus *Polerovirus*, family *Solemoviridae*) in canola [77]. For the currently less important virus host combinations in Table 3, in most instances the yield loss data provided comes from outside Australia as relevant Australian yield loss data are lacking.

## 3. Cereal Viruses

Two yellow dwarf viruses (YDVs), BYDV and cereal yellow dwarf virus (CYDV; genus *Polerovirus*, family *Solemoviridae*), and WSMV cause diseases that threaten wheat, barley, oat and triticale crops in Australia [78,79,80,81,82,83,84,85,86,87,88,89,90,91,92]. They cause the most important cereal crop virus diseases in the continent’s Mediterranean and temperate climatic regions (Figure 1; Table 2). Johnsongrass mosaic virus (JGMV) infects Australia’s maize, sweet corn, pearl millet and sorghum crops across northern Australia, causing their commonest and most economically significant virus disease [93,94,95,96]. As numerous Australian studies over the last 40 years have demonstrated, YDVs cause considerable yield losses in cereals growing in southern Australia [69,70,71,72,73,90,97,98,99]. Fahim et al. [74] demonstrated yield losses of 22 to 44% from WSMV in wheat in south-east Australia, and an 83% yield reduction was recorded in a fully infected wheat crop compared to the average yield in adjacent healthy wheat crops in south-west Australia [87,100]. In addition, yield losses of similar magnitude were recorded in the major WSMV epidemic of 2005 in graze-grain wheat in the slope region of NSW [101,102]. When severe JGMV symptoms develop in infected maize and sorghum crops, grain yield losses can reach similarly high levels in Australia [75,76]. The following section describes past Australian research on BYDV, CYDV, WSMV and JGMV in cereals. It then summarizes Australian research on the other nine other viruses found so far infecting cereals in the continent (Table 3) and makes recommendations for future research.

### 3.1. Yellow Dwarf Viruses

**Figure 3:**
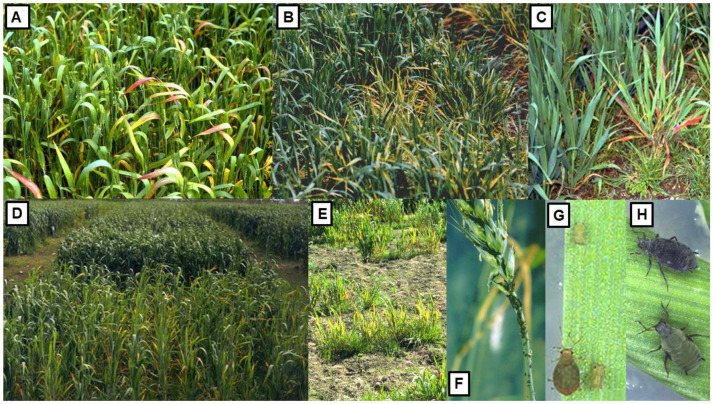
YDV symptoms in cereals, and cereal aphid vector, images from Australia. (**A**), Wheat crop with YDV-infected plants showing reddening and yellowing of flag leaves and lower leaves. (**B**), Barley crop showing a patch of YDV-infected plants (bottom right) and healthy more vigorous plants (top left and center). (**C**), Oat crop with YDV-infected plant showing reddening of lower and chlorosis of upper leaves and stunting (right), other oat plants healthy and vigorous. (**D**), Untreated control plot of wheat containing stunted YDV-infected plants with chlorosis and reddening of leaves (front), insecticide-treated plot containing vigorous healthy plants (behind). (**E**), Single row plots in YDV resistance evaluation trial showing infected wheat plants with leaf yellowing and reddening and severe stunting. (**F**), Heading tiller of wheat heavily colonized by the YDV vector *Rhopalosiphum padi*. (**G**), An adult wingless *R. padi* with two young offspring. (**H**), Two adult wingless adults of the YDV vector *R. maidis*. (**G**,**H**) image credits @Department of Primary Industries and Rural Affairs/D. Thackray. Images (**A**,**E**) modified from [8].

The first Australian study on YDV’s was published in 1957 [91]. Over the six decades since then, they have been the subject of much Australian research. They are phloem-limited viruses spread from plant-to-plant by aphid vectors that transmit them persistently but are not transmitted through seed [103]. The symptoms they elicit in different cereal species in Australia vary in severity depending on crop species infected, cultivar sensitivity, virus strain, growth stage when infection occurs and environmental conditions. They include interveinal chlorosis, yellowing or reddening of leaves, an upright posture, stiff leaves, stunted growth, delayed or reduced heading and shriveled grain (Figure 3A–E), and both root length and biomass are decreased [13,79,83,84,86].

In southern Australia, *Rhopalosiphum padi* (Figure 3F,G; bird cherry-oat aphid) is the most common YDV vector whereas *R. maidis* (Figure 3H; corn leaf aphid) is the second commonest [70,72,79,98,104,105,106]. By contrast, in south-east QLD and tropical northern Australia, *R. maidis* is much commoner than *R. padi* [83,107]. In addition, five other aphid vector species infest Australian cereal crops and sometimes play minor roles in spreading YDV’s [35,105]. They are *Metopolophium dirhodum* (rose-grain aphid), *R. insertum* (apple-grass aphid), *R. rufiabdominalis* (rice root aphid), *Sitobion miscanthi* (wheat aphid), *S. fragariae* (blackberry-cereal aphid) [70,72,79,83,84,98,106,108,109,110]. *M. dirhodum* is restricted to south-east Australia and SA [108,111]. In TAS, *S. miscanthi* is less common than *S. fragariae* [112], but the opposite situation mostly occurs in mainland Australia [109].

Currently, 11 YDV variants are recognized worldwide [113,114]. However, the only variants found in Australia so far are: BYDV-PAV, BYDV-MAV, CYDV-RPV and maize yellow dwarf virus (MYDV-RMV) [35,79,80,81,83,86,89,110,115]. Parry et al. [35] summarised the information available up until 2012 about their distribution within wheat crops in different parts of Australia. BYDV-PAV was the commonest and CYDV-RPV the second commonest. The incidences of BYDV-PAV or CYDV-RPV infected plants recorded in cereal crops varies from region to region, and both frequently occur in mixed infections [79,80,84,86,89]. The highest YDV incidences found in cereal crops were in south-west Australia, where 65% and 39% of samples were infected with BYDV-PAV and CYDV-RPV, respectively [84]. BYDV-MAV and MYDV-RMV both sometimes infect cereals in southern Australia, but generally occur at lower incidences than BYDV-PAV or CYDV-RPV [80,81,85,110,115,116]. However, in south-east QLD MYDV-RMV was the commonest YDV variant infecting grasses and YDV incidences were lower overall than in the rest of Australia in wheat crops [83].

In early studies in North America, YDV variants were subdivided based on their aphid vector species: BYDV-PAV was transmitted by *R. padi* and the *Sitobion* species *S. avenae* (grain aphid), BYDV-MAV by *S. avenae*, MYDV-RMV by *R. maidis* and CYDV-RPV by *R. padi* [117]. Although *S. avenae* is absent from Australia, *S. miscanthi* and *S. fragariae* are both present (see above). In Australia, Sward and Lister [81] reported that *M. dirhodum* and *S. miscanthi* transmitted BYDV-MAV and BYDV-PAV at moderate or low efficiencies, respectively, and Guy [85] reported that *M. dirhodum* transmitted BYDV-PAV and CYDV-RPV from mixed infections containing both of them. Guy et al. [79] reported that *S. fragariae* transmitted BYDV-PAV on its own, and, occasionally, CYDV-RPV too when present in mixed BYDV-PAV and CYDV-RPV infections, and De Barro [118] reported that it transmitted BYDV-PAV at low efficiency. Therefore, in southern Australia, the more frequent occurrence of BYDV-PAV and CYDV-RPV infections in cereal crops than of infections with BYDV-MAV and MYDV-RMV correlates with a greater abundance of *R. padi* than any of the six other aphid vector species [35]. By contrast, the abundance of *R. maidis* in south-east QLD [83] likely explains why MYDV-RMV is the commonest YDV variant there.

Since the original extensive surveys for YDV’s in cereal crops around southern Australia, the only studies to establish more up-to-date information on YDV incidences in Australian crops are two in the south-east of the continent. When small numbers of preserved wheat samples obtained from the NSW ‘slopes region’ in 2006–2013 were tested, BYDV-PAV was detected every year, and CYDV-RPV in 2012 and 2013 [119]. In 2014, a severe YDV epidemic occurred in wheat, barley and oats, 73% of samples collected being infected by YDV’s. In samples without mixed YDV infections the overall infection incidences were: 27% (BYDV-MAV), 19% (BYDV-PAV) and 14% (CYDV-RPV). When wheat and barley crops were sampled annually over a 4-year period (2014–2017) across western VIC, an unexpectedly high percentage of crops and plants were found infected; the overall incidences of infected plants were 9% with BYDV-PAV, 5% with BYDV-MAV and 3% with CYDV-RPV [89]. Out of 83 cereal crops assessed, 75% were YDV-infected and the maximum incidence at sampling time was 58%. Although taking samples at different stages of crop development might have been a contributing factor to the different incidences reported, the mean BYDV-PAV incidence of 9% across all regions and fields represents a more than four-fold increase over the overall BYDV-PAV incidences found in a previous study conducted in the same region 30 years previously [80]. Moreover, this figure included those for a low rainfall region (the Mallee) where the YDV occurrence was considerably smaller than in the other two regions sampled (the Wimmera and south-west). Given that under Australian conditions yield losses increase by about one percent with every 1% increase in BYDV-PAV incidence [69,72,73], average annual yield losses of 9% through 2014–2017 were attributed to BYDV-PAV. Additionally, estimated yield losses in some individual fields were >50% in some years based on the virus incidence levels. Recent field experiments in VIC revealed yield losses of up to 80% and 64% in wheat and barley, respectively, from BYDV when infection occurred at early crop growth stages. Such losses were largely attributed to a decrease in grain number, likely a result of a reduction in plant growth. However, yield losses varied considerably between experiments highlighting the importance of other variables such as climate and agronomy in determining yield loss outcomes [73]. Field experiments in south-west Australia found that early BYDV-PAV infection in wheat caused 44–64% yield losses at the same site in different years, and significantly decreased seed weight by 88% and protein content by 69% [72]. For each 1% virus incidence increase, a yield decrease of 55–72 kg/ha occurred. Earlier field experiments with wheat also demonstrated significantly decreased seed size following early BYDV infection [70,71,98]. By contrast, apart from sporadic yield losses that sometimes occurred in spring, it proved difficult to demonstrate any losses in wheat yields arising from direct aphid feeding damage [120].

Over a 30-year period (1983–2001), extensive surveys found infection with YDV’s to be widespread in wild and pasture grass species throughout southern Australia and in TAS, including in their grainbelt regions. BYDV-PAV, BYDV-MAV, CYDV-RPV and MYDV-RMV were all found in these studies, but their relative incidences varied with grass species, rainfall zone and region [35,79,81,83,84,85,86,105,110,112,115,116,121,122]. In south-east QLD, BYDV-PAV, BYDV-MAV and MYDV-RMV were present in pasture and wild grasses but CYDV-RPV was absent [82,83]. Jones [31] combined all this YDV host data listing more than 50 wild and pasture grass species acting as YDV hosts from within Australia’s different climatic zones including samples taken at different seasons of the year from diverse habitats. Cereal volunteer plants were also YDV hosts. Since aphid vector species also colonized these same grass species and volunteer cereals, they constitute a very widespread and substantial reservoir from which aphid vectors can spread YDV’s to cereal crops, especially in grainbelt high rainfall cropping zones. This source also enables YDV’s and aphid vectors to survive in between cereal growing seasons in different parts of Australia [35,105,116,123,124].

In Australian grain growing regions with Mediterranean climates, such as south-west Australia, both YDV’s and the aphids that transmit them survive the dry summer and early autumn period in perennial grass hosts and volunteer cereal plants that persist in damp places. These places are normally roadside ditches kept moist by dew runoff, the edges of creeks, wet areas around grain silos or water troughs, springs and irrigated gardens [116]. The principal drivers of YDV epidemics and the yield losses they cause are: (i) size of the nearby YDV infection and cereal aphid reservoir; (ii) time of arrival of infective aphid vectors in crops; (iii) aphid vector activity within crops; and (iv) climatic factors (temperature, rainfall and wind) that influence aphid numbers, movement and virus transmission [72,124]. Initial aphid arrival time in crops was related closely to the amount of late summer and early autumn (March–April) rainfall prior to sowing. Soil moisture resulting from rainfall events promotes growth of volunteer cereal plants and grass weeds, creating a ‘green bridge or ramp’ where aphids multiply before their flights to autumn-sown crops take place. Lack of adequate rainfall results in insufficient plants being available to enable aphid survival before sowing, which means that they end up arriving in cereal crops much later [72,116,124]. Apart from along its southern coastline, perennial pastures that might act as BYDV and aphid vector reservoirs over the dry summer period are lacking in the south-west Australian grainbelt. However, they are present in some parts of south-east Australia [35]. Only a very small area of irrigated dairy pasture in the vicinity of Harvey, which is outside the south-west grainbelt, and sheep pastures sown with the perennial grass *Pennisetum clandestinum* (Kikuyu grass) along the grainbelt’s southern coastline, allow YDV’s and their aphid vectors to persist over-summer [84,104,110,121]. Although they may sometimes be important close to the south coast, these perennial pasture YDV sources are currently far too small to play any role in initiating YDV epidemics in cereal crops across the broad expanse of the south-west grainbelt. Nevertheless, in the future, climate change induced global warming and increased rainfall over summer is likely to increase aphid populations and YDV alternative host survival in some Australian grainbelt regions causing increasingly severe YDV epidemics (see Section 5.3 below).

Field experiments in south-west Australia demonstrated that applying two early sprays of a pyrethroid insecticide to kill aphid vectors was very effective at suppressing YDV spread in cereal crops. Moreover, treating seed with neonicotinoid insecticide and delaying sowing to avoid the peak autumn cereal aphid flight period were also effective in controlling it, as was avoiding the peak autumn cereal aphid flight period by delayed sowing [70,98]. Therefore, insecticide treatments involving seed treatment and foliar insecticide applications were widely adopted as control measures. They were applied prophylactically regardless of whether there was risk of early aphid arrival and widespread infection. Therefore, a forecasting model and decision support system was developed to alert farmers before sowing time each year about whether the risk of losses from YDV’s in their locality was too low to justify insecticide use [124]. Its wide-scale adoption in south-west Australia awaits automation of the weather data collection for the model. Also, before it can be used in parts of south-east Australia that lack a Mediterranean-type climate, it may require modifications that consider local environmental and other factors, such as widespread presence of perennial grasses in perennial pastures, that allow YDV’s and their aphid vectors to persist during summer. Meanwhile, widespread prophylactic seed dressing and foliar insecticide applications has greatly reduced YDV incidence in south-west Australian cereal crops. However, widespread YDV infection remains a significant future threat because of: (i) the likelihood of a neonicotinoid insecticide ban being enacted; (ii) neonicotinoid insecticide resistance developing locally in *R. padi*; and (iii) the arrival in Australia of the YDV vector *S. avenae*, which already has a broad insecticide resistance profile [125,126].

Over the last 40 years, producing new barley and wheat cultivars with resistance to BYDV-PAV has been a major focus of Australian cereal breeding programs [99,127,128,129,130,131,132,133,134,135,136,137,138]. With barley, the first resistance gene found was *Ryd1*, which came from barley cultivar ‘Rojo’ [139], but it confers minimal YDV resistance so has rarely been used in breeding. The second and third barley resistance genes found were *Ryd2* and *Ryd3*, both originally from Ethiopian barley germplasm, which confer resistance to BYDV-PAV and BYDV-MAV by reducing virus titer [140,141]. A fourth resistance gene, *Ryd4*, which confers more effective resistance to BYDV-PAV, was introduced to barley from the wild species *Hordeum bulbosum* [142]. More recently, two major QTL’s for CYDV-RPV resistance were discovered in a population of recombinant inbred barley lines [143]. To date, gene *Ryd2* has been used widely in breeding Australian barley cultivars for BYDV resistance, although combining *Ryd2* and *Ryd3* confers greater resistance than either gene on its own [127,129,136,138]. In the future, combining QTL’s that confer CYDV-RPV resistance with *Ryd* genes that give resistance to BYDV-PAV and BYDV-MAV would be needed to breed Australian barley cultivars with more effective YDV resistance. With wheat, four resistance genes are available for use in breeding new wheat cultivars for YDV resistance, *Bdv1*, *Bdv2*, *Bdv3* and *Bdv4*. *Bdv1* comes from a Brazilian wheat cultivar and wheat germplasm. It confers partial resistance to BYDV-MAV by reducing virus titer. *Bdv2*, *Bdv3* and *Bdv4* are all from intermediate wheatgrass (*Thinopyrum intermedium*). They confer partial resistance to BYDV-PAV (*Bdv2*), full resistance to CYDV-RPV and moderate resistance to BYDV-MAV and BYDV-PAV (*Bdv3*), or partial resistance to BYDV-PAV (*Bdv4*) [114,128,133,136]. In Australia, *Byd2* has been widely used to breed BYDV-PAV resistant wheat cultivars [144], but resistance genes *Bdv1*, *Bdv3* and *Bdv4* are now all being used currently in wheat breeding [137]. In the future, combining *Byd2* with *Bdv3* seems highly desirable as a wheat breeding objective, because it would confer resistance to the two most widespread YDV’s in Australia, BYDV-PAV and CYDV-RPV. Genetic modification to achieve YDV resistance in cereals has received little attention in Australia, but one study found a virus-derived transgene-encoding hairpin RNA gave immunity to BYDV-PAV but not to CYDV-RPV in barley [145].

The glasshouse screening procedure recently developed to evaluate Australian barley and wheat germplasm for resistance to BVDV-PAV [134], should also be used to reveal whether resistance to BYDV-MAV and CYDV-RPV is present. Field evaluation for YDV resistance involving spread from nearby natural infection reservoirs should always include testing of leaf samples by ELISA or other methods to establish which YDV’s are present. Currently, in Australia the extent of infection recorded is often based on visual observation for foliage symptoms, all the symptomatic plants being assumed to be BYDV-PAV infected [144]. This assumption is likely to provide misleading results, especially when CYDV-RPV is present [146].

### 3.2. Wheat Streak Mosaic Virus

In 2002, WSMV was found in wheat breeding facilities in the Australian Capital Territory [147], and soon afterwards was shown by Australian research to be seed-borne in wheat [100,148,149]. In 2003, it appeared in a small number of irrigated wheat crops and wheat breeding sites in NSW, and at other predominantly wheat breeding sites in SA, VIC and QLD. In 2004, it was reported again occurring at low incidence in several wheat crops in NSW. By 2005–2006, WSMV infection was widespread in wheat crops in the ‘slopes area’ of the southern NSW high-rainfall zone in the south-eastern wheatbelt. Over 5000 ha (2005) and 20,000 ha (2006) of early sown ‘graze-grain’ crops were devastated by these outbreaks [101,150]. In 2006, it appeared in the south-west Australian grainbelt, occurring in all of its rainfall zones. Incidences were up to 100% in some crops [87,100]. When preserved wheat samples from the NSW ‘slopes region’ were tested, WSMV was detected in samples from 2006–2011: infection being greatest in 2006, 2008 and 2009 when 38%, 42% and 50% of samples were infected, respectively [119]. More recently in south-west Australia, thorough use of herbicide sprays to kill volunteer cereals and grasses before sowing wheat had resulted in relatively few infected crops suffering significant WSMV yield impact in most years [88,146]. However, WSMV infection remains a major limitation to Australian dual purpose (graze and grain) wheat systems in which it causes considerable forage and grain yield losses [87,88,100,149].

**Figure 4:**
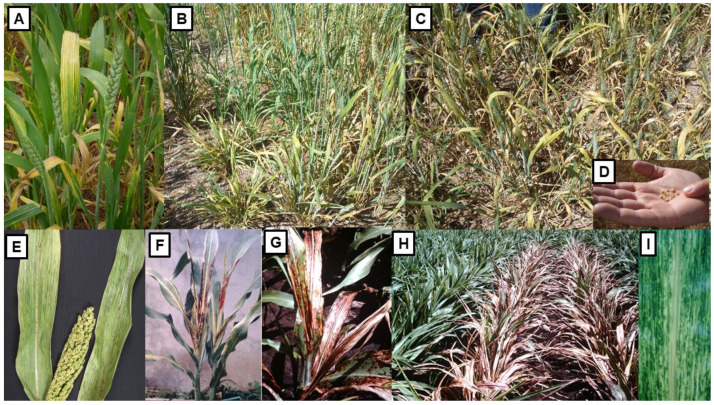
Symptoms of wheat streak mosaic virus (WSMV) in wheat, and Johnsongrass mosaic virus (JGMV) in sorghum and maize in Australia. (**A**) WSMV-infected wheat plants showing yellowing and green streaking of flag leaves. (**B**) WSMV-infected wheat crop with two early infected plants showing dwarfed and stunted growth with chlorotic yellowed leaves (front left) and a mixture of other plants showing yellow-leaf symptoms or lacking symptoms. (**C**) WSMV-infected crop showing wheat plants with reduced tillering, weak stems, and yellowed foliage. (**D**) Shriveled, small seeds harvested from a WSMV-infected wheat crop (indicated by finger). (**E**) JGMV–infected sorghum plant showing mosaic symptoms in flag leaves. (**F**) JGMV–infected sorghum plant with ‘red stripe’ leaf symptoms. (**G**) JGMV–infected sorghum plant with necrotic symptoms. (**H**) Two rows of sorghum plants with leaves killed by JGMV-infection (right), rows of healthy plants (left). (**I**) JGMV-infected maize leaf showing mosaic symptoms. Images (**A**,**C**) modified from [8].

The foliage symptoms WSMV infection causes in wheat in Australia include pale green streaking of leaves, yellowing of older leaves and stunted, tufted plant growth (Figure 4A–C). Early infection dwarfs growth, causes fewer tillers to form, and results in poor seed set and filling, decreased grain size and weight, and shriveled grain (Figure 4D) [87,88,100,149]. Sequencing of representative isolates from around Australia suggested wheat seed carrying a WSMV strain from USA’s Pacific Northwest probably arrived 10–20 years before its first detection in 2002, possibly at the Tamworth via Post Entry Quarantine (PEQ) facility in NSW. Its mite vector was already present in Australia. WSMV was then spread around the continent inadvertently in infected wheat seed reaching the south-west Australian grainbelt after a considerable delay [150]. At the time of its initial introduction, visual inspection was the mandated screening method for quarantinable cereal viruses, and failure to intercept it in PEQ was likely due to lack of familiarity with its foliar symptoms or asymptomatic infection.

The WSMV vector is *Aceria tosichella* (the wheat curl mite, WCM), a tiny, wingless eriophyid mite found throughout the Australian grainbelt [151,152]. WCM transmits WSMV semi-persistently [153], spreading it when infective mites carried by the wind reach healthy plants [87,88]. In an extensive survey to detect its presence in alternative hosts surviving outside the cereal growing season in isolated wet spots in the south-west Australian grainbelt, WSMV was found occurring at very low incidences in three annual wild or pasture grass species [31,100]. When Coutts et al. [88] inoculated plants of wheat, oat and barley cultivars, and of both perennial and annual grass species often present in the south-west Australian grainbelt, WSMV infected all three cereals and all annual grass species, but no perennial grass species. Seedlings were-grown from seeds collected from these infected plants, but wheat was the only species in which seed transmission occurred, being detected at 0.03–0.1% infection levels. WSMV-infected seedlings survived sufficiently well to develop into plants that formed viable seeds and for WCM to acquire and transmit WSMV to healthy plants [88,148,150].

In field experiments with wheat in which different numbers WSMV-infected and WCM-infested plants were introduced to simulate 0.1–1.4% seed-borne WSMV infection within plots, WSMV’s final incidence increased from 17 to 97% depending upon the number of infector plants present [88]. Coutts et al. [88] concluded that in Australian grainbelt regions with Mediterranean climates where rainfed cereal crops must be sown in autumn and harvested in spring, sown infected seed stocks and seed-infected volunteer wheat seedlings play a crucial role in initiating new WSMV epidemics. This is because, due to in the dry, hot summer conditions between wheat crops, significant alternative WSMV infection reservoirs are lacking so WSMV can only persist in this period in spilt wheat seed or stored wheat seed stocks. When wheat seed is sown and volunteer plants germinate from the infected wheat seeds, the infected wheat seedlings act as initial WSMV infection foci for WCM to acquire and spread WSMV in the wheat crop [8,87,88]. This component of WSMV’s disease cycle, where seed infection is of crucial importance, contrasts with the situation in North America’s Great Plains region where annual autumn wheat sowings shortly after wheat crops are harvested permit WCM to transmit WSMV from residual infected volunteer cereal plants to young wheat seedlings [8,88,154]. Other factors that predispose Australian wheat crops to WSMV epidemics, include warm growing season conditions that encourage rapid build-up of its WCM vector and extended growing seasons, such as those involved in graze-grain cropping system [8,87,88]. In the future, climate change induced global warming and increased rainfall over summer are likely to enhance WCM populations and alternative host survival in grainbelt regions with Mediterranean climates causing increasingly severe WSMV epidemics (See Section 5.3 below).

WSMV research for Australian wheat breeding programs has focused on using both conventional and molecular genetic modification approaches to develop new wheat cultivars with resistance to this virus and to WCM [74,155,156,157,158,159]. In glasshouse experiments, the resistances conferred by genes *Wsm1* originally from *Thinopyrum intermedium* and *Wsm2* from wheat breeding line C0960293-2, and the resistance present in wheat breeding line c2652 were all effective against WSMV [156]. However, *Wsm1* and most *Wsm2* derivatives were temperature sensitive being ineffective above 20 °C, whereas breeding line c2652, *Wsm2* selection CA745 and several amphiploids were still resistant at 20–28 °C. Several molecular markers were devised for use in breeding for WSMV resistance [156]. In field experiments, Fahim et al. [74] found that *Wsm1*, *Wsm2*, *Wsm2* derivatives and breeding line c2652 all protected wheat against WSMV infection, and Richardson et al. [159] found potentially useful new sources of WCM resistance in *Th. intermedium*, *Th. ponticum* and *Hordeum marinum*. Two Australian studies explored genetic modification approaches to generate WSMV-resistant wheat. When an RNA interference (RNAi) construct consisting of hairpin RNA derived from the nuclear inclusion protein ‘a’ (*NIa*) gene of WSMV was introduced, it conferred immunity to this virus in transgenic wheat plants [155]. Similarly, when an artificial miRNA strategy targeting conserved regions of WSMV was used, some transgenic wheat plants immune to WSMV were obtained [157,158].

In Australian research, two protocols were developed for sensitive real-time qRT-PCR detection of WSMV in bulk wheat seed samples: (i) for direct seed sample testing and (ii) for seedling testing [146,160,161]. Protocol (i) detected surface WSMV seed contamination so provided a measure of the proportion of seeds that come from WSMV-infected mother plants. Protocol (ii) detected the seed embryo infection with WSMV that gives rise to infected wheat seedlings in the field so it provided a measure of the WSMV infection foci that will result in the crop when infected seed stocks of wheat are sown. Because of the low level of WSMV embryo infection that occurs (in around 0.2% of seeds), the values obtained for seed infection from (i) greatly exceed those from (ii). Both methods were highly sensitive, e.g., with (ii) up to 5000 seedlings can be tested together in a single extraction. Farmers receive warning from (i) about WSMV presence in their seed stocks, and from (ii) about the likely epidemiological significance of the seed infection present.

Based upon the understanding of factors driving WSMV epidemics in Australia and how to manage them described above, and WSMV control measures developed earlier in North America e.g., [154], the following integrated disease management approach was recommended for WSMV control in parts of Australia with Mediterranean climates:—“(i) remove all potential WSMV and WCM hosts prior to crop sowing to ensure there is no inoculum for spread to the new wheat crop; (ii) sow late when temperatures are low and not conductive to WCM population build up or movement; (iii) plant WSMV-resistant wheat cultivars if available; (iv) sow healthy wheat seed after getting a seed sample tested before sowing; and (v) ensure no spilt wheat seed is left in the field after harvest of infected crops” [87,88]. To achieve (v), harvesters adapted to collect and exclude small seeds from the main seed harvest can be used, as occurs with weed seed. However, (ii) will become less effective over time due to the more erratic climate caused by climate change (see Section 5.3 below).

More information is required about the epidemiology of WSMV under Australian grainbelt conditions. For example, knowledge is lacking over its possible transmission by contact, as occurs in field pea with pea seed-borne mosaic virus [162], and whether wind-assisted contact transmission occurs within the growing wheat crop. Such transmission would result in enlargement of infection foci derived from seed-infected wheat plants such that the virus source for its acquisition and spread would be greater once WCM become active. Studies are also lacking over how well WSMV survives on surfaces (e.g., metal or rubber) and which disinfectants could be used effectively to remove surface contamination with its particles. Field experiments to quantify the seed yield and quality losses WSMV causes also need to be completed, and an epidemiological model and Decision Support System for WSMV is required.

### 3.3. Johnsongrass Mosaic Virus

For more than 30 years, JGMV was the subject of much research in Australia, and between 1968 and 1998 many scientific papers were published on this subject. Initially, JGMV was described as ‘Australian maize dwarf mosaic virus’ [163], then as the Australian Johnsongrass strain of sugarcane mosaic virus (SCMV-Jg) [164], and, finally, it was considered sufficiently distinct to be considered a separate virus [165,166]. JGMV is a non-persistently aphid-borne virus, and its known aphid vectors are *Aphis craccivora*, *Aphis gossypii*, *Myzus persicae* and *R. maidis* [164]. It infects grain and fodder sorghum, maize, sweet corn, pearl millet, Johnsongrass (*Sorghum halepense*), wild sorghum (*Sorghum vertricilliflorum*), and other species of *Poaceae* [165]. Shepherd and Holdeman [167] suggested a low level of JGMV seed transmission in maize and sweet corn but did not include suitable healthy controls to provide greater confidence in the results. In addition, there were no known reference virus isolates from these experiments to obtain genome sequence to confirm JGMV was used. JGMV is common in eastern subtropical (NSW and QLD) regions of Australia, being most common and damaging in sorghum crops. It also occurs across the entire tropical northern (WA, NT and QLD) and south-eastern regions of Australia Table 2 [13,93,94,95,96,164,168,169,170].

Two strains of JGMV are recorded in Australia, the type strain (JGMV-JG) and the Krish-infecting strain (JGMV-K) which differs in being able to infect sorghum cultivars with the Krish resistance gene [171,172,173,174]. JGMV occurs frequently in cereal crops because Johnsongrass, a naturalized perennial grass and its principal host, occurs commonly in Australia, especially in the wetter parts of the coastal and sub-coastal regions of NSW and south-east QLD [169,175]. This grass species allows JGMV to survive readily between cereal growing seasons, and acts as a major reservoir for aphids and virus to spread to sorghum, maize, sweet corn and pearl millet crops. In sorghum, it causes three main types of symptoms, ‘mosaic’ consisting of leaf mosaic often restricted to young leaves (Figure 4E), ‘red stripe’ consisting of red and necrotic stripes along leaves (Figure 4F), and ‘red leaf’ which starts as typical mosaic but develops into severe necrosis at lower temperatures (Figure 4G,H) [76,93,94,176]. Symptom expression in sorghum is under genetic control. Mosaic symptoms are the most common across genotypes. The necrotic red stripe symptom is controlled by a dominant *N* gene and occurs at both high and low temperatures while the necrotic red leaf symptom is expressed only under low temperatures in infected plants with the recessive *rlf* gene [93,177]. In maize and sweet corn, JGMV causes ‘maize dwarf mosaic disease’ consisting of mosaic, ringspots, chlorosis and stunting (Figure 4I) [75,95,168,169]. In pearl millet, it causes mosaics of varying severity and stunting, and in a sensitive cultivar that developed severe symptoms it caused a 60% decrease in foliage dry matter yield [96]. In field experiments with sorghum, grain yield losses due to JGMV were over 50% in sensitive cultivars that developed red stripe and red leaf symptoms, both seed size and number being reduced. These yield losses reached 92% in an inbred line showing severe leaf reddening. Moreover, up to 25% losses were still observed in tolerant cultivars that developed mild symptoms [76]. In maize, the yields of susceptible hybrids were diminished by up to 50% and JGMV infection caused almost total crop failure in inbred lines [75].

Australian research on controlling JGMV has focused on resistance breeding in sorghum and maize [76,93,94,95,171,172,173,178,179,180]. Resistance to JGMV was found in the Indian sorghum cultivar Krish [171,172,173,174,180], and sorghum breeding line Q7539 [94,180]. The single dominant resistance gene from Krish sorghum was incorporated into parental lines the QLD sorghum breeding program used to breed new hybrid cultivars [176]. However, a virus strain designated JGMV-K, overcame this resistance gene [172,174], which was attributed to a two amino acid difference in the virus coat protein [181]. Many sorghum cultivars produced by this breeding program carried this resistance gene. However, they developed severe symptoms following infection with JGMV-K which soon became the dominant strain in QLD [176]. Therefore, the QLD breeding program’s sorghum germplasm collection was screened for resistance to JGMV-K. Breeding from crosses made with JGMV-resistant sorghum breeding line Q7539 [94,180] provided progenies with a high level of resistance and the progeny plants that did become infected but only developed mild symptoms [176]. The resistance in breeding line QL19 derived from Q7539, performed well when exposed to JGMV-K in the field [176]. In maize, the first JGMV resistance study involved demonstrating presence of high levels of resistance in open pollinated cultivars [178]. Next, when in-bred maize lines from Australia and elsewhere were evaluated for JGMV-resistance by sap inoculation and field exposure, seven were highly JGMV-resistant to both, and nine to field exposure [95]. When the progeny of crosses using JGMV-resistant maize breeding line KL57 as a parent were inoculated with JGMV, the segregation ratios obtained were consistent with single gene JGMV-resistance. No resistance to JGMV was found in 15 pearl millet accessions tested in Australia [96].

JGMV has had less impact on Australian sorghum and maize crops in the last 20 years for two reasons. Firstly, roadside and on-farm hygiene to minimize populations of Johnsongrass has improved [165], and secondly Australian sorghum cultivars with JGMV resistance have been grown widely [176]. Also, most of the sweet corn cultivars being grown in Australia are from the USA where they are bred for maize dwarf mosaic virus (MDMV, genus *Potyvirus*; family *Potyviridae*) resistance, which confers resistance to JGMV. Hence, these imported sweet corn cultivars have JGMV resistance [182,183]. However, JGMV has continued to be detected at low levels in maize and sorghum in south-east QLD and northern NSW, and an increase in crop infections has occurred recently (M. Sharman, unpublished data). These sporadic detections may be due to incomplete JGMV resistance in some cultivars, geographical differences in the level of virus in nearby alternative hosts or the appearance of a new resistance breaking virus strain. Research to monitor currently occurring JGMV strains and the resistance status of newly released cultivars is required to determine the current level of risk from JGMV-induced losses. Genetic modification to achieve JGMV resistance in cereals has received little attention in Australia, but one study attempted to achieve transgenic resistance using the coat protein and large nuclear inclusion protein coding sequences of JGMV to transform sorghum [179].

The possibility that a low-level of seed transmission of JGMV occurs in maize [167], and potentially also in sorghum, should be tested with Australian isolates to determine if it plays a role in JGMV’s epidemiology. Although in the past most Australian JGMV disease outbreaks were attributed to crops being grown near infected Johnsongrass, the possibility that low-level carry-over by seed-borne infection occurs between growing seasons, creating primary infection foci within crops sown with infected seed stocks requires investigation. If seed-transmission of JGMV were to be confirmed in maize or sorghum, then healthy seed stocks and seed health testing could be important not only in avoiding movement of JGMV to new regions but also in reducing within-crop spread from infected seedlings acting as primary infection foci within crops. Since many years have elapsed since JGMV strains were studied in Australia, and both grain cultivars and farming practices have changed considerably since then, a revised assessment of the geographical distribution of JGMV strains and the JGMV resistance of modern sorghum and maize cultivars is needed to determine the current risk to grain production from infection with this virus. In addition, field studies on the spatial and temporal dynamics of JGMV spread in sorghum, maize and pearl millet fields and the annual patterns of JGMV aphid vector activity are lacking as are studies field studies on any phytosanitary and cultural control measures, apart from removal of Johnsongrass growing near crops. An epidemiological model and decision support system for JGMV is also required to help ensure its effective management when severe outbreaks occur.

### 3.4. Other Cereal Viruses

For each of the nine other viruses found infecting cereal crops in Australia, information on their nomenclature, the crops they infect, their vectors, the foliage symptoms, seed yield losses and seed quality defects they cause, whether they are seed-borne and their occurrence in Australia is provided in Table 3. High plains wheat mosaic virus (HPWMoV), previously known as high plains virus, wheat mosaic virus or maize red stripe virus, resembles WSMV in being transmitted by WCM and causing similar foliage symptoms in wheat (see Section 3.2 above). It has been recorded occurring at low incidences in wheat crops in south-west and south-east Australia [119,184]. Australian HPWMoV isolates had nucleotide sequence identities very similar to those of a Nebraska (USA) isolate [184]. Seed-transmission of HPWMoV at low levels is reported in sweet corn [185,186], so may also occur in wheat which would explain it occurrence in wheat parts of Australia where maize and sweet corn are absent. Three strains of SCMV, which is non-persistently aphid-transmitted, are present in tropical and subtropical Australia: SCMV-SG, SCMV-Q and SCMV-SC. Their main natural hosts are sugarcane for SCMV-SC, sabi grass (*Urochloa mosambicensis*) for SCMV-SG and Queensland blue couch grass (*Digitaria didactyla*) for SCMV-Q [164]. SCMV-Q occasionally spreads to maize and sweet corn in the field, but SCMV-SG and SCMV-SC do not, despite all three strains infecting sorghum following sap inoculation [95,164,169]. There are no reports of them infecting pearl millet or Johnsongrass in the field in Australia. In addition, as mentioned above (in Section 3.1), aphid-borne MYDV-RMV, which is transmitted by *R. maidis* and occurs most commonly in subtropical QLD [83], sometimes infects cereal crops (barley, oats, wheat) in Australia’s different grainbelt regions. There is also a record of the aphid-borne virus barley virus G infecting barley wheat, oats and triticale in south-east Australia [187]. In addition, there are occasional records from subtropical east, Mediterranean, and temperate regions of Australia of barley being infected with the contact and seed-borne virus, barley stripe mosaic virus [13,188,189]. In tropical and subtropical eastern Australia, the maize sterile stunt strain of planthopper transmitted barley yellow striate mosaic virus infects maize and sweet corn [13,190], and two leafhopper transmitted viruses, cereal chlorotic mottle virus and chloris striate mosaic virus, infect barley, oats, maize, sweet corn, wheat and rye [13,191]. In addition, a planthopper transmitted virus, maize stripe virus, infects maize, sweet corn and sorghum in these same Australian regions [13,192].

### 3.5. Possible Occurrence of Soil-Borne Cereal Viruses

The plasmodiophorid protist *Polymyxa graminis* is an obligate parasite of cereal roots. It is an important vector of viruses which, between them, damage wheat, barley, oats, triticale, rye, maize, sweet corn, pearl millet, sorghum and rice crops in other parts of the world, where they cause substantial yield losses (see Section 5.1 below). Its motile zoospores swim in soil moisture and spread soil-borne cereal viruses from roots of infected plants to roots of healthy wheat plants, and its resting spores survive for many years in soil in the absence of cereal crops [193,194]. In 2009, *P. graminis* was detected infecting barley roots in southern QLD and identified as *P. graminis* f. sp. *tepida* [193]. In 2011–2012, *P. graminis* was found in samples of wheat roots from the south-west Australian grainbelt and three WA isolates were classed as *P. graminis* f. sp. *temperata* (ribotypes Ia and Ib), and three as f. sp. *tepida* (ribotypes IIa and IIb) [194]. Both *P. graminis* f. sps transmit soil-borne viruses to cereal crops. However, when samples from wheat crops in the WA wheatbelt were tested by RT-PCR for soil-borne wheat mosaic virus (SBWMV; genus *Furovirus,* family *Virgaviridae*), soil-borne cereal mosaic virus (SBCMV; genus *Furovirus*, family *Virgaviridae*), wheat spindle streak mosaic virus (WSSMV; genus *Bymovirus*, family *Potyviridae*), and for furoviruses in general, no virus infection was detected. This suggests two *P. graminis* introductions to Australia, and the presence of two f. sps suggests soil-borne cereal viruses may well become established soon, if not already present [194]. Such establishment of a soil-borne cereal virus, along with *P. graminis,* has already occurred in fields in the South Island of New Zealand where SBWMV is now present most likely having arrived with imported cereal seed contaminated with viruliferous resting spores of *P. graminis* f. sp. *temperata* resting spores [195].

### 3.6. Recommendations for Further Research

With YDVs’s, the forecasting model and decision support system of Thackray et al. [124] awaits automation of weather data collection before it can be deployed in south-west Australia. In addition, its deployment in south-eastern Australia awaits its modification to take into account local environmental, cultural and other factors that differ from those found in the south-west. Moreover, an important aim of Australian cereal breeding should be to breed new barley, oats and wheat cultivars with comprehensive YDV resistance. To achieve this, the glasshouse and field procedures used for large-scale routine screening for YDV resistance in cereal germplasm and breeding lines need to be expanded from the current focus on BYDV-PAV so that they also include screening for BYDV-MAV and, especially, CYDV-RPV resistance. This will ensure that resistance to BYDV-MAV and CYDV-RPV are also present.

With WSMV, an important component of its epidemiology that still requires investigation is whether it is contact-transmitted and whether wind-assisted contact transmission occurs within the growing wheat crop. Also, large-scale field experiments are needed to quantify the yield losses it causes under different Australian WSMV epidemic scenarios. To help manage it more effectively studies are needed to establish how well its virus particles survive on surfaces and which disinfectants are suitable for surface contamination removal. In addition, an epidemiological model and decision support system is required to ensure effective management when a serious WSMV outbreak is likely to occur in wheat crops.

While there have not been major outbreaks of JGMV in sorghum or maize for many years, an increase in crop infections has occurred recently so this situation may change. To protect not only these two economically important crops, but also crops of sweet corn and pearl millet, there are several knowledge gaps that need to be addressed. No JGMV resistance screening of Australian bred maize or sorghum cultivars has been done for many years so there is a major gap in our knowledge concerning the resistance status of currently grown commercial cultivars. Also, a recent survey to establish the spectrum of JGMV strains now present across Australia’s maize, sweet corn, sorghum and pearl millet growing regions, and which strains are most common in cereal crops and alternative hosts, is lacking. Such a survey would identify which isolates need to be used in JGMV resistance screening and whether any recent crop infections are being caused by a resistance-breaking JGMV strain. In addition, the possibility that low-level seed transmission may play a role in its epidemiology should be tested with Australian JGMV isolates. Moreover, some other aspects of its epidemiology and management in sorghum, maize, sweet corn and pearl millet crops have yet to be studied in Australia (see Section 3.3 above) so this situation needs to be rectified. In addition, an epidemiological model and decision support system for JGMV is required.

Further research is required to understand how changing circumstances, especially alterations in farming systems and climate, are likely to influence the epidemiology and management of virus disease epidemics in Australian cereal crops in the future. Such research is needed to prepare for more severe cereal virus disease epidemics, especially those caused by YDV’s, WSMV, and JGMV. Addressing these epidemics will require conducting research on (i) the effects of climate change (extreme weather events, insufficient or excessive rainfall, increased temperature and wind speed) and altered farming system parameters upon virus spread, and (ii) addressing the increasing difficulties in controlling them.

Research is needed to understand the pathogenicity and genetic variability of YDV, WSMV and JGMV isolates from Australia’s different grain growing regions. This includes comparing their virulence in different cereals and obtaining their genomic sequences to establish their diversity.

Since there is no information over the important subject of virus diseases that afflict rice in Australia, this constitutes an important knowledge gap that needs to be addressed. The same applies to soil-borne cereal viruses. A thorough national survey to establish their occurrence is required.

## 4. Oilseed Viruses

Over the past decade, Australia has produced up to 3.5 million tonnes of oilseed per annum, of which canola accounts for over half and cottonseed about a third, with smaller contributions from soybean, sunflower, safflower, peanut, Indian mustard, linseed and sesame [196]. Amongst these oilseed crops, sunflower, linseed, sesame, and peanut serve a dual purpose as their edible seeds are also consumed directly by humans. In addition, cottonseed, soybean and Indian mustard serve as a multi-purpose crops as they are used to produce fiber and feed livestock (cottonseed), feed humans and livestock (soybean), or feed humans and provide biofuel (Indian mustard) [197]. In Australia, the most serious virus disease of oilseed brassicas is caused by TuYV. However, due to the appearance of a resistance breaking strain that overcomes the turnip mosaic virus (TuMV; genus *Potyvirus*, family *Potyviridae*) resistances present in Australian canola cultivars, the second most serious is caused by TuMV [26,77,198,199,200,201,202,203,204,205,206,207,208,209,210,211,212,213,214,215]. Both are important in the continent’s Mediterranean, temperate and subtropical climatic regions (Figure 1; Table 2). Field experiments with canola in south-west Australia demonstrated a seed yield loss up to 46% caused by TuYV [77]. In a glasshouse experiment in NSW, infecting a mixture of mustard and canola plants, with TuYV or TuMV diminished their seed yields by 12% or 84% overall, respectively [205]. The most significant virus diseases of sunflower and cotton are caused by tobacco streak virus in sunflower (TSV; genus *Ilarvirus*, family *Bromoviridae*) [216,217,218,219,220], and cotton bunchy top virus in cotton (CBTV; genus, *Polerovirus*, family *Solemoviridae*) [147,221,222,223,224]. They are important in Australia’s subtropics and are present in its tropical regions (Figure 1; Table 2). TSV reduced yields by 70% in sunflower [217,218] and CBTV by 67% in cotton [222]. The following section describes past Australian research on TuYV and TuMV in canola, TSV in sunflower and CBTV in cotton. It then summarizes Australian research on the other 14 viruses found so far infecting oilseed crops in the continent (Table 3) and makes recommendations for future research.

### 4.1. Turnip Yellows Virus

Over the last 40 years, TuYV has been the subject of research in Australia (Table 2). It was first reported in 1982 in TAS under the name beet western yellows virus BWYV; [225]. Thereafter in the 1980s, in addition to being studied in depth in TAS [29,198,226,227,228], it was found in NSW, QLD and WA [13,229]. During the 1990s, there were no further TuYV studies until surveys in 1998–1999 revealed its widespread occurrence in canola crops in the south-west grainbelt [199]. Research interest therefore resumed, and after combining the published detections in natural hosts from the 1980s with those published in 2000–2021, it is now known to occur commonly in the south-west, south-east and north-east Australian grainbelt regions and TAS, and to also be present in the tropical north. Furthermore, TuYV not only infects crops and weeds in the *Brassicaceae*, but also a wide range of other species belonging to different plant families. These natural hosts include crop volunteer, pasture and weed species in the *Amaranthaceae*, *Apiaceae*, *Asteraceae*, *Boraginaceae*, *Cucurbitacae*, *Euphorbiaceae*, *Fabaceae*, *Geraniaceae*, *Malvaceae*, *Oxalidaceae*, *Portulacaceae, Rhizophoraceae*, *Solanaceae* and *Tropaeolaceae* [13,25,26,27,28,29,30,77,199,201,203,206,207,211,212,213,214,230,231,232,233,234,235,236,237,238,239,240].

**Figure 5:**
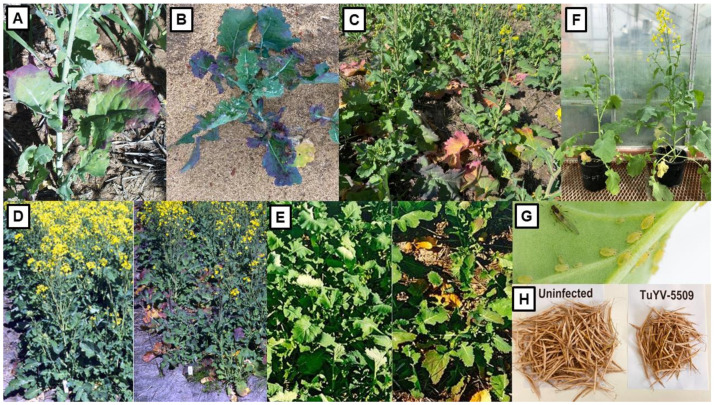
Turnip yellows virus infection in canola plants in Australia. (**A**) Reddening of lower leaves. (**B**) Purpling of lower and middle leaves. (**C**) Reddening, chlorosis, premature senescence and leaf drop of lower leaves. (**D**) Untreated (left) and insecticide treated (right) plots in canola yield loss field experiment: untreated plot with stunted, spindly plant growth and lower leaves with reddening and purpling, premature senescence and leaf drop versus treated plot with healthy vigorous plant growth versus. (**E**) Untreated (left) and insecticide treated (right) plots with early infection in canola yieldloss field experiment: untreated plot with young plants showing reddening, chlorosis, early senescence, and leaf drop versus treated plot with healthy young plants. (**F**) Infected (left) and uninfected (right) plants in pot experiment: infected plant with stunted growth but asymptomatic leaves versus uninfected plant with vigorous healthy growth. (**G**) *Myzus persicae* vector: winged adult (top left) and its wingless young offspring (right). (**H**) Seed pod harvests from individual infected (right) and uninfected (left) plants in yield loss pot experiment: pods from infected plant are smaller and contain fewer seeds. (**G**) image credit @Department of Primary Industries and Regional Development/P. Scanlon.

The foliage symptoms TuYV causes in infected canola crops include yellowing, reddening or purpling, subsequent premature senescence of lower leaves, and plant stunting (Figure 5A–D). As infected plants grow taller, this coloration can spread to some middle leaves, and shoot growth becomes ‘spindly’ with diminished branching and flowerin resulting in a reduction in seed pods. An infected crop may begin flowering earlier than expected and its canopy appear uneven. In addition, symptoms tend to be most severe when young canola crops become infected shortly after seedling emergence. In these scenarios, patches of stunted seedlings with severe leaf discoloration and plant dwarfing can develop, and some seedlings may die (Figure 5E) [77]. When infection occurs later, less severe symptoms develop (Figure 5F) [77,203]. However, canola cultivars differ in sensitivity to TuYV infection, the more tolerant ones developing mild symptoms or even asymptomatic infection [203]. Environmental factors, especially temperature, are also likely to influence symptom expression. Unfortunately, farmers and agricultural advisers often incorrectly attribute TuYV symptoms in canola plants to nutrient deficiencies or other abiotic stresses.

In 2002, TuYV was distinguished from BWYV due to host range differences [241] and partially sequenced south-west Australian isolate WA-1 was found to resemble TuYV soon afterwards [77,201]. However, because both viruses always reacted strongly with BWYV antiserum in serological tests involving Australian isolates, the name BWYV continued being used [77,201,203,236]. This changed in 2012 when Wilson et al. [237] found partially sequenced Tasmanian BWYV isolates all resembled isolate WA-1 in grouping with TuYV, and therefore commenced using the name TuYV for Australian ‘BWYV’ isolates. In subsequent studies, complete genomic nucleotide sequences of south-west Australian isolates [214], followed by isolates from VIC, SA, NSW and QLD [239], all belonged to TuYV rather than BWYV. A further study revealed the existence of three distinct genetic variants of TuYV [240].

TuYV is a phloem-limited, persistently aphid-borne virus that can be transmitted by at least 15 aphid species [13,242]. In Australian canola crops, *M. persicae* (green peach aphid) is the principal aphid vector of TuYV due to its high transmission efficiency (>90%) and propensity to rapidly colonize vast areas of the crop. *Brevicoryne brassicae* (cabbage aphid) was shown to transmit TuYV in Europe [242]. Although no Australian TuYV transmission experiments have been done with *B. brassicae*, it is only considered a minor vector in Australia due to its relatively low recorded transmission efficiency and habit of localized, dense vertical colonization, limiting the extent it spreads this virus to other plants [77,201]. There are no records suggesting that the other main aphid colonizer of canola in Australia, *Lipaphis pseudobrassicae* (turnip aphid), can transmit TuYV, although it was shown to transmit a New Zealand BWYV isolate that might have been TuYV [243]. TuYV is not known to be seed-borne or transmitted by contact [13]. In addition to infecting canola and Indian mustard, other cultivated plant species it infects in Australia include diverse vegetable and forage brassicas, crop and pasture legumes, and sugar beet (*Beta vulgaris*), lettuce (*Lactuca sativa*), spinach (*Spinacia oleracea*), potato (*Solanum tuberosum*), melon (*Cucumis melo*), coriander (*Coriandrum sativum*) and parsley (*Petroselinum crispum*) [13,25,26,27,28,29,30,200,201,203,206,214,230,232,233,236,237,239]. Amongst the many wild Australian hosts within different plant families found infected with TuYV mentioned above (see first paragraph in this Section), the most common infection reservoirs for its spread to oilseed crops include: *Raphanus raphanistrum* (wild radish), *Rapistrum rugosum* (turnip weed), *Myagrum perfoliatum* (muskweed), *Malva parviflora* (marshmallow) and *Sonchus oleraceous* (common sowthistle) [199,201,206,230,231,236]. Their relative importance as TuYV infection reservoirs for spread to crops varies with region and climate, e.g., *R. raphanistrum* is critically important in the south-west and the south-east, *M. perfoliatum* and *R. rugosum* in south-east [199,230,236], and *M. parviflora* in the subtropical east [231].

When large-scale surveys to establish the incidence of TuYV infection in canola crops in south-west Australia were done, it was detected in 59% and 66% of crops sampled in 1998 and 1999, respectively [199]. At time of sampling, the incidences of TuYV infection ranged from 1 to 65% within individual infected crops in both seasons. TuYV was most frequently found infecting canola crops in high and medium rainfall zones but also found often in the low rainfall zone. When samples of *R. raphanistrum* were tested, TuYV was found in all three rainfall zones with infection incidences of up to 96%. Similarly, when *R. raphanistrum* samples from seven high rainfall sites were tested in 2001, it was detected at six of them with incidences of up to 65% [230]. Both studies revealed a widespread TuYV infection reservoir present in this common alternative weed host. In 2002, an average infection incidence of 41% was reported for 37 canola crops in NSW [200,205]. In 2013, TuYV was detected in all canola crops sampled in northern NSW, reaching an incidence of 93% within individual infected crops [206]. In 2014, a TuYV epidemic occurred in canola crops in the grainbelt regions of SA, western VIC and southern NSW, resulting in seed yields being severely diminished [234]. When 618 samples from these regions were tested, 57% were TuYV-infected, with the highest numbers of detections in those from SA (87% positive). In 2015, TuYV was the most important virus infecting canola in south-east Australia [207]. In 2016, it was detected in 92% of dual-purpose canola crops sampled in western VIC, with an average in-crop incidence of 47% and individual crop incidences of up to 92% at time of sampling [244]. In 2018, a severe TuYV epidemic developed in canola crops in the Esperance region of south-west Australia with infection incidences within many individual crops reaching >60% before flowering [238,245]. Thus, in recent growing seasons, Australia has experienced relatively frequent and severe TuYV epidemics across all main areas of its canola production.

In field experiments with a sensitive canola cultivar in south-west Australia, rapid TuYV spread at the most vulnerable early crop growth stage caused the greatest seed yield losses and impaired seed quality. Due to a reduction in seed number, seed yield losses reached 46%, and for every 1% increase in TuYV incidence, yield was diminished by 6–12 kg/ha. Furthermore, TuYV infection was associated with a significant decrease in seed oil content and an increase in erucic acid content [77]. In a glasshouse experiment in which TuYV was aphid-inoculated to plants belonging to canola cv. Ag Outback, and five breeding lines and one cultivar of Indian mustard, TuYV diminished their seed yields by an average of 12% across all the mustard and canola plants [205]. In further glasshouse experiments, plants of an open-pollinated and a hybrid canola cultivar were aphid-inoculated with TuYV at four different growth stages; two leaf, seven leaf, beginning of stem elongation and full flowering [212]. In both cultivars, substantial seed yield losses (26 to 41%) occurred following inoculation at the two leaf and seven leaf growth stages. However, when inoculated at the beginning of stem elongation, substatial seed yield losses (26%) only occurred in the open-pollinated cultivar indicating tolerance in the hybrid cultivar at this growth stage. No losses occurred when plants of either cultivar were inoculated at full flowering time. In the severe TuYV epidemic in SA in 2014, widespread early infections in canola crops were estimated to have caused yield losses of up to 75% [234]. Therefore, TuYV can cause substantial economic damage, particularly to sensitive canola cultivars, and when it reaches high incidences during vegetative growth stages. Additional biotic and abiotic stressors are likely to impact the magnitude of seed yield losses associated with TuYV infection. For example, there might be differences in virulence between the three genetic variants of TuYV identified by Filardo et al. [240], but this has yet to be studied.

During the dry summer periods of 2000, 2001 and 2002, surveys of over 10,000 plants growing across the entire south-west Australian grainbelt found TuYV persisted in plants of volunteer canola and four common broad-leafed weed species (*Citrullus lanatus* var. *citroides*, *Conzya* spp., *Navarretia squarrosa* and *Solanum nigrum*). The infected plants were surviving in isolated wet locations, mostly roadside verges, usually near culverts or within roadside ditches in all three grainbelt regions (north, central, south) and two of its rainfall zones (high, medium) [201]. Moreover, small populations of the two known TuYV canola vector species (*M. persicae, B. brassicae*) were present in all three rainfall zones, mostly infesting volunteer canola plants and the weeds *R. raphanistrum* or *S. oleraceous*.

The size of TuYV epidemics and the consequent yield losses in canola crops vary between years, seasons, rainfall zones, and geographic regions [235]. What was the explanation of how a small reservoir of TuYV and its vectors surviving outside the growing season vectors can be sufficient to initiate major TuYV epidemics in canola crops in some years? The answer was revealed using (i) canola data collection blocks set up annually over a 3-years (2000–2002) at four representative grainbelt sites [246,247,248], and (ii) information from three other field studies on the TuYV-canola pathosystem [77,199,201]. The main factors were the size of the external virus infection source; its proximity to canola crops; time of first arrival, abundance, and activity of *M. persicae* vectors; the sowing date and length of the growing season; environmental factors including rainfall, temperature and wind that affect aphid behavior, aphid numbers, and virus transmission; and crop plant density [235]. As with YDVs in this region (see Section 3.1. above), initial aphid arrival time in canola crops was related to the volume of late summer and early autumn (March–April) rainfall prior to sowing. Soil moisture from rainfall events allowed volunteer crop plants and weeds to grow forming a ‘green bridge or ramp’ in which aphids multiplied before flying to autumn-sown crops. Inadequate rainfall resulted in insufficient plant growth to enable large-scale aphid build-up before sowing, which meant that vector aphids arrived much later in canola crops [77,201,235]. This knowledge was used to develop an epidemiological model for externally acquired TuYV in canola crops. The model successfully predicted TuYV spread for 10 out of the 12 datasets from the data collection blocks [235]. However, since the period when the field data upon which this model was developed was collected, the time of sowing of canola crops across southern Australia has moved from late-autumn to early- to mid-autumn when the temperature is warmer [249]. This means that the extent of aphid activity during early to mid-autumn plays a critical role in initiating TuYV epidemics in canola crops. Loop-mediated isothermal amplification (LAMP) testing of flying aphids caught on sticky traps placed at multiple south-west Australian grainbelt locations over two years (2017–2018), found that the magnitude of virus-carrying winged aphids caught between sowing time and the five-leaf growth stage was correlated with infection incidence by stem elongation. The sticky trapping approach provided an early warning system for TuYV epidemics and is currently being utilized across the WA, VIC and NSW canola growing regions [211,238,245]. The data collected from this system over the past five growing seasons would be valuable in re-calibrating the forecasting model to take into account changes since 2010, including identifying other drivers of autumn aphid activity. Furthermore, the effects on TuYV epidemic development arising from climate change induced alterations in growing season temperature and rainfall patterns in Australian grainbelt regions would need to be incorporated [6,9,39,250].

Australian studies on TuYV management in canola crops have focused mainly on insecticide seed treatment application to protect vulnerable seedlings from early *M. persicae* colonization and identifying host resistance [77,203,211,213]. Farmers have relied on insecticides (particularly neonicotinoid seed treatments) to control *M. persicae* and TuYV in canola crops. However, there are several serious future threats to their effective deployment, and these are discussed in Section 5.2 below. In 2001–2002 south-west Australian field experiments, applying the neonicotinoid imidacloprid to canola seed at twice the recommended commercial rate before sowing provided very effective control of *M. persicae* vectors, suppressed TuYV spread for up to 10 weeks and increased canola seed yield by 84–88% [77]. However, in 2003–2005 further field experiments using imidacloprid applied at the recommended commercial rate obtained inadequate suppression of both aphid vectors and TuYV [77,251]. A likely explanation was provided in glasshouse tests when viruliferous *M. persicae* were placed on canola seedlings grown from commercially treated canola seed [203]. The aphids infested 72% of seedlings and transmitted TuYV to 62% of them indicating that most seeds were inadequately coated with insecticide. Improved procedures for the commercial seed dressing process were therefore recommended. After 2008, canola seed treatment with neonicotinoids increased rapidly in Australia [252]. However, in 2017, de Little et al. [253] found reduced sensitivity (conferred by low-level metabolic resistance) to neonicotinoids in Australian *M. persicae* populations which is now widespread and could be diminishing the efficacy of neonicotinoid seed treatments. Foliar application of insecticides to control *M. persicae* is limited due to insecticide resistance in this aphid species, particularly against carbamates, organophosphates and synthetic pyrethroids (see Section 5.2 below). As recently as 2020, foliar insecticidal control of *M. persicae* in canola was restricted to application of sulfoxaflor, but likely due to over-reliance on it and its misuse, reduced sensitivity to it emerged in *M. persicae* in Australia [254]. In 2021, foliar insecticides with two different active ingredients, afidopyropen and flonicamid, were registered for control of *M. persicae* in Australian canola crops [255]. Whether such foliar insecticides are effective at reducing TuYV spread is yet to be established. As discussed in the preceding paragraph, an early warning system that detects viruliferous aphids migrating into canola crops [211,238], combined with re-calibrating the forecasting model of [235], could enable non-prophylactic, targeted insecticide applications, thereby avoiding their unnecessary overuse.

In 2006, when 18 commercial cultivars of canola were evaluated for TuYV resistance by exposure to field infection in south-west Australia, three of them (Stubby, Trigold, Tranby) had useful infection resistance (I^R^ = resistance to transmission by infective aphids), and this was confirmed via aphid-inoculations in the glasshouse [203]. During the TuYV epidemic season of 2014 in south-eastern Australia, the Australian canola cultivar Stingray never developed foliage symptoms in infected commercial crops. Furthermore, except at one site where the infection rate was higher, Stingray developed fewer symptomatic plants and markedly lower infection rates in field experiments at multiple sites in SA and NSW [234,256]. Between 2017 and 2020, more extensive field and glasshouse evaluation for TuYV resistance included 43 Australian canola cultivars (including Stingray), 100 *B. napus* accessions obtained from the ERANET-ASSYST diversity set [257], six *B. napus* genotypes with known resistance (including in Stubby, Trigold and Tranby progenitors) and 36 vegetable brassica cultivars. Of the commercial canola cultivars, Stingray had moderate I^R^ but stronger I^R^ was found in the ERANET-ASSYST diversity set canola accessions Liraspa-A, SWU Chinese 3 and SWU Chinese 5 [213]. In addition, resistance to virus accumulation (=multiplication) (A^R^) often accompanied I^R^. Furthermore, very strong A^R^ and moderate I^R^ was identified in several *B. oleracea* cultivars. However, unlike A^R^, I^R^ was temperature-sensitive as, although effective at 16 °C, it was much less effective at 26 °C and ineffective at 30 °C [213]. Research to identify the genetic drivers of these resistance sources is required. Furthermore, all resistance sources need to be challenged with the broad genetic spectrum of Australian TuYV isolates to identify any strain-specificity. Canola breeders have already successfully incorporated A^R^ and I^R^ into European canola cultivars (R54-derived resistance) which provide seed yield benefits compared to susceptible cultivars under high disease pressure conditions [258]. Therefore, with the current uncertainties concerning future insecticidal management of *M. persicae* and TuYV discussed above and in Section 5.2 below, developing commercial TuYV-resistant cultivars should be a high priority for Australian canola breeders.

The integrated disease management approach recommended to control TuYV infection of canola crops in south-west Australia involves combining phytosanitary, cultural, chemical and host resistance measures taken at or just before sowing time [203,235,255]. These measures are: (i) applying herbicide at least 2 weeks before sowing (phytosanitary); (ii) manipulating the sowing date (cultural); (iii) sowing at high seeding rates or narrow row spacing (cultural); (iv) sowing in standing stubble (cultural); (v) dressing canola seed with insecticide (chemical); and (vi) planting TuYV-resistant cultivars (host resistance) (see Table 1 for explanation of how these control measures operate) [203,234,235]. Amongst these measures, (i) is already a standard phytosanitary practice for removing the green bridge in and around fields prior to sowing [56,59], (v) has already been adopted widely and based on field experimentation with neonicotinoid insecticides [77], and (vi) depends on development of suitable high yielding, locally adapted TuYV resistant canola cultivars. Cultural measures (ii), (iii) and (iv) are based on field experimentation with pathosystems involving non-persistently aphid-borne viruses [33,56,59] so whether they can decrease TuYV spread in canola crops warrants future investigation. However, field observations made during the 2014 epidemic in southern Australia suggested that TuYV incidences were lower at sites with standing stubble, high plant density and later sowing [234]. The wide scale adoption of such cultural measures will also depend upon their compatibility with current agronomic practices (see Section 5.5 below).

### 4.2. Turnip Mosaic Virus

**Figure 6:**
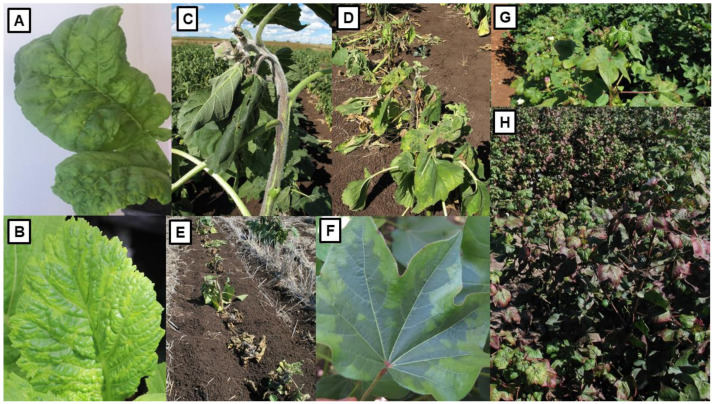
Symptoms of turnip mosaic virus (TuMV) in canola and Indian mustard, tobacco streak virus (TSV) in sunflower and cotton bunchy top virus (CBTV) in cotton in Australia. (**A**) TuMV-infected canola leaves with mosaic symptoms. (**B**) TuMV-infected Indian mustard leaf with mosaic symptoms. (**C**) TSV-infected sunflower plant with necrosis (black streaking) of the leaf lamina, petiole and stem, shortened internodes and a weakened stem. (**D**) Sunflower plants collapsing due TSV-infection. (**E**) Row of dead and dying sunflower plants caused by TSV-infection. (**F**) Leaf of CBTV-infected cotton showing pale light-green angular patterns along leaf margins. (**G**) Young shoot of a CBTV-infected cotton plant with leaf symptoms of downward cupping, marginal curling, deformation and reduction in size (in foreground). (**H**) CBTV-infected mature cotton crop showing reddening and downcurling of their leathery textured upper leaves. (**A**,**B**) image credit @University of Western Australia/E. Nyalugwe. Images (**A**,**B**) modified from [265] and [208], respectively.

TuMV was first reported in Australia infecting three vegetable brassica crops (broccoli, cauliflower, and cabbage; *B. oleracea*) in NSW in 1959 [259], and again in cabbage in 1975 [260]. By 1988, it had been found in all Australian states (NSW, QLD, SA, TAS, VIC, WA), but not yet in the NT (Table 2) [13]. The crops found infected in these early Australian studies included canola, vegetable brassicas (broccoli, cabbage, cauliflower), turnip (*B. rapa* subsp. *rapa*), watercress (*Nasturtium officinale*), wallflower (*Erysimum cheiri*), common stock (*Matthiola incana*) (all *Brassicaceae*), lettuce, and nasturtium (*Tropaeolum majus*). In addition, it was found infecting three common *Brassicaceae* weeds, *Hirschfeldia incana* (Buchan weed), *Sinapsis arvensis* (wild mustard), *R. rugosum* (wild turnip) [13]. Little further attention was given to TuMV in Australia until 1998–2001, when it was reported infecting canola, Indian mustard and rocket (*Eruca vesicaria*), the non-*Brassicaceae* crop rhubarb (*Rheum rhabarbarum*), and again in the important *Brassicaceae* weeds *R. raphanistrum, R. rugosum, S. arvensis*, and *Sisymbrium* sp. [199,200,205,261]. TuMV causes a diverse range of symptoms that depend on interactions between the virus strain present, host plant, and environmental conditions. The foliage symptoms it causes in plants of infected Australian canola cultivars include leaf mosaic (Figure 6A), deformation and necrosis, and plant stunting or death [202,208,209,210,262,263]. Those that develop in infected Indian mustard plants include leaf mosaic (Figure 6B), crinkling, vein banding and chlorosis, necrosis, reduced podding, plant stunting and premature plant death [204,205,209,210,215,264,265]. In a glasshouse experiment in NSW in which TuMV was inoculated to plants belonging to five breeding lines and one cultivar of Indian mustard, and to TuMV-susceptible canola cv. Outback, it diminished seed yields by 84% overall [205].

In 1998–1999, surveys in south-west Australia found TuMV infection in 5% of 139 (1998) and 2% of 47 (1999) canola crops sampled [199]. The infection incidences were 1–5% in infected crops. TuMV was present in three out of 10 cultivars and occurred in high and medium rainfall grainbelt zones. It was also present in the weed species *R. raphanistrum* in both this survey and a 2001 survey in the horticultural region adjacent to the high rainfall zone [230]. In surveys in NSW in 1999–2007, TuMV was found infecting the *Brassicaceae* crops Indian mustard and forage turnip, the grain legume crops chickpea, and field pea and the *Apiaceae* crop coriander. It was also present infecting the *Brassicaceae* weeds mentioned in the previous paragraph along with *Fumaria* sp. No natural infection was detected in canola samples, but the incidence of TuMV in experimental plots of Indian mustard reached 25% in 2002, 55% in 2003, and almost 100% in 1999 [200,205]. In 2016, TuMV was detected in six of 12 spring-sown dual-purpose canola crops sampled in western VIC, with an average in-crop incidence of 2% at time of sampling [244]. These low levels of infection within TuMV-infected canola crops from different parts of Australia reflect the frequent presence of TuMV resistance genes in Australian canola cultivars described in detail later in this Section. By contrast, in the Liverpool Plains region of NSW, TuMV was found causing widespread infection in two canola crops in 2012 and in canola crops belonging to several cultivars in 2013 reaching high incidences [206]. In earlier UK studies using four *B. napus* differentials consisting of canola and swede lines to distinguish them, 12 distinct pathotypes were identified amongst TuMV isolates from around the world [266]. Presence of a resistance-breaking pathotype in NSW which broke all the TuMV-resistances in Australian canola cultivars, was confirmed by Guerret et al. [210]. Thus, when isolates 12.1 and 12.5 of this pathotype and one each of pathotypes 1 (NSW-2), 7 (NSW-2) and 8 (WA-Ap1) were inoculated to plants of 19 Australian cultivars and one breeding line of canola, only 12.1 and 12.5 always overcame their TuMV resistances. Although this pathotype has spread to canola crops in southern NSW (J. van Leur, personal communication), follow up large-scale surveys to determine the extent of its spread from the Liverpool plains to canola crops growing in other parts of Australia are lacking. This seems largely due to the severe drought years that followed in eastern Australia (especially in 2017 and 2019). Because of the high yield limiting potential of TuMV in canola crops (see previous paragraph), and its detrimental effect on stored canola seed viability [262], this is something that urgently needs to be done.

In Australia, TuMV is transmitted non-persistently by the *Brassicaceae* aphid colonizing species *M. persicae*, *B. brassicae* and *L. pseudobrassicae* [13,77,201], and, potentially, by many other aphid species that do not colonize canola but visit the crop whilst searching for their preferred hosts [205,263]. It is not known to be seed-borne [13]. Frequently infected common weed hosts that survive outside the oilseed crop growing season in grainbelt regions, include the weeds *R. raphanistrum* in south-west Australia, and *R. rugosum*, *S. arvensis* and *Sisymbrium* sp. in NSW [199,205]. These hosts enable TuMV to persist between successive annual sowings, and during the growing season aphid vectors spread it from them to susceptible crops. Studies with 27 complete genomes of TuMV isolates from around Australia found that the Australian sequences were in phylogroups I and II of World-B, II of Basal-BR and IV of Basal-B [209,210,267]. They occurred at four different positions within World-B, two within Basal-B and one within Basal-BR so at least seven separate introductions of different genetic variants are present. Resistance-breaking pathotype isolates 12.1 and 12.5 grouped separately from two other Australian sequences (AUST19 and AUST23) within phylogroup II of World-B [210]. Thus, there is knowledge of (i) TuMV’s spread from plant-to-plant by aphid vectors that transmit it non-persistently, (ii) the many alternative crop and weed hosts likely to act as reservoirs for spread to canola and Indian mustard crops (see previous two paragraphs), and (iii) its considerable biological and genetic diversity within Australia. Otherwise, the epidemiology of TuMV in Australian oilseed crops is yet to be investigated.

When 43 Australian canola cultivars or breeding lines were inoculated with TuMV pathotype 8 isolate WA-Ap, only one cultivar (Outback) was susceptible (phenotype +) [202]. With one exception where two plants developed phenotype R_N_/+ (localized necrosis with systemic spread without necrosis), the other 42 lines all developed one or more of three different resistance phenotypes: R_N_ (localized necrosis without systemic spread) which was the commonest, +_N_ (systemic movement with necrosis), and R (localized resistance to systemic movement without necrosis). As expected for an outcrossing species, some cultivars or lines (22) segregated for 2–3 different types of resistance phenotype (+_N_, R and/or R_N_). Phenotype O (extreme resistance) was absent [202]. In the first of two subsequent Australian studies, plants of 99 canola accessions, breeding lines and cultivars from Australia and three other world regions were inoculated with TuMV pathotype 8 isolate WA-Ap1 [265]. Only two cultivars from Australia (cvs Muster and Scoop), were uniformly susceptible (phenotype +), and only two other lines developed resistance phenotype +_N_. The other 95 lines developed one or more of resistance phenotypes O, R and R_N_, with R_N_ again occurring most often. In the study referred to previously in the second paragraph of this section, resistance-breaking isolates 12.1 and 12.5 always elicited phenotype + in 19 Australian canola cultivars and one breeding line [210]. In contrast, when inoculated with isolates WA-1 (pathotype 8), NSW-1 (pathotype 7) and NSW-2 (pathotype 1), four different resistance phenotypes (O, R_N_, R, and +_N_) developed either singly or segregating in different combinations, none of them developing susceptible phenotype + [210]. Interpreting the resistance phenotypes found in terms of different canola TuMV resistance genes is complicated [210]. Phenotype +_N_ develops with pathotype 3 when dominant gene *TuRB01b* is present [268,269,270]. When dominant genes *TuRB01* and *TuRB03* are present, phenotype O develops with pathotypes 1 and 3, respectively. When these genes occur singly with some TuMV isolates (pathotypes unspecified), phenotype O develops with dominant gene *TuRB04* and phenotype R_N_ with dominant gene *TuRB05*. However, *TuRB04* is epistatic to *TuRB05,* and, when both occur together, phenotype O develops with pathotype 3. Phenotype R develops when dominant gene *ConTR01* and recessive gene *retro01* coincide with pathotypes 1, 3–4, 7–9 or 12 [268,269,270]. Phenotype R_N_/+ does not correspond with any known resistance gene [202,210,265]. Therefore, since only pathotypes 1, 7 and 8 were used (pathotype 3 unavailable), the frequent finding of phenotypes R_N_ and O may indicate the presence of *TuRB01* and *TuRB03*, and, more frequently, of *TuRB04* and *TuRB05*. The less common presence of phenotype R may indicate the less frequent occurrence of *ConTR01* and *retro01.* Since pathotype 3 was absent, phenotype +_N_ cannot be attributed to gene *TuRB01b* so may have elicited an unknown resistance gene. The protection that these diverse resistance genes offer against TuMV infection explains the low incidences of this virus recorded in Australian canola crops before 2012, after which the situation began to change due to the appearance of its resistance breaking strain in NSW (see previous paragraph).

When TuMV pathotype 8 isolate WA-Ap1 was inoculated to 44 accessions, breeding lines and cultivars of Indian mustard, five of black mustard (*B. nigra*) and nine of Ethiopian mustard (*B. carinata*) available in Australia, a diverse spectrum of TuMV resistance phenotypes developed [204]. These resembled those found previously in canola or were variants differing in severity. Those that developed in Indian mustard and *B. nigra* included ones resembling phenotype +_N_, and others that resembled phenotype R_N_. However, the former were often associated with premature plant death (phenotype +_ND_) and the latter with phenotype R_N_/+. Phenotypes R and O were absent, only one line developed phenotype + uniformly (cv. Tendergreen), and segregation for different phenotypes occurred commonly. By contrast, the resistance phenotypes that formed in the nine Ethiopian mustard lines were R, R_N_ and O, and these always prevented systemic infection. Segregation for 2–3 of these resistance phenotypes occurred in all but one of them, but phenotypes +_N_, +_ND_, R_N_/+ and + were absent. When a selection of lines belonging to all three species were inoculated with isolates belonging to pathotypes 1 (NSW-2), 7 (NSW-1) and 8 (WA-Ap1), the phenotypes were similar, indicating they were unrelated to differences in pathotype [204]. When the same TuMV pathotype 8 isolate was inoculated to 32 lines of Ethiopian mustard germplasm, phenotype O developed in 14, phenotype R_N_ or R in two each, and one was uniformly phenotype + [265]. In the other lines, there was segregation for phenotypes O and R or R_N_ and R. In a further study, 69 more germplasm lines of Indian mustard were inoculated with the same TuMV pathotype 1 isolate [208]. The results were similar to those found previously with this species, as resistance phenotypes +_N_, +_ND_, R_N_/+ and a variant of the latter developed. Only 22 lines developed a single phenotype uniformly which was phenotype + in just one of them. Amongst these, the 11 lines from Australia developed phenotypes R_N_/+, +_N_, +_ND_ and +. In the 47 lines with phenotypic segregation, there were 2–3 different phenotypes in each of them. In addition, when six lines were each inoculated with the same three pathotype 1, 7 and 8 isolates, the resistance phenotypes were again unrelated to differences in pathotype [208].

When F2 progeny plants of the Indian mustard cross JM 06006 (+ only) and Oasis CI (+_ND_ only) were inoculated with pathotype 8 isolate WA-Ap1, the segregation ratios obtained fitted a 3:1 ratio (systemic necrosis: susceptibility) at an early stage of infection, but the ratio was 1:2:1 (+_ND_: +_N_: +) at a late stage of infection [208]. This suggested a single incompletely dominant resistance gene named *TuRBJu01* controlling expression of phenotypes +_ND_ and +_N_ in the homozygous and heterozygous conditions, respectively. When plants of parent Oasis CI, which carries *TuRBJu01,* were held at 16° and 28 °C after inoculation, they developed phenotype +_ND_ at both temperatures suggesting *TuRBJu01* is not temperature sensitive. Plants of the two parents were challenged by inoculation with Australian isolates from pathotypes 1 (NSW-2, NSW-6), 7 (NSW-1, NSW-5) and 8 (WA-Ap, WA-Ap1), along with isolates of unnamed new pathotype(s) (NSW-3, NSW-4), and of canola resistance-breaking pathotype (12.1, 12.5) [264]. Parent Oasis CI developed phenotype +_ND_ with all isolates except NSW-3, whereas parent JM 06006 developed susceptible phenotype + with all 10 isolates. When F3 progeny plants were challenged by inoculation with all isolates apart from NSW-3, apart from with isolates 12.1 and 12.3, all fitted a 3:1 ratio well at an early stage of infection. With 12.5 and 12.1, a good fit was absent. Therefore, *TuRBJu01* is strain-specific as it was less effective against the canola resistance-breaking pathotype and ineffective against NSW-3 [264]. In contrast to the frequent occurrence of resistance phenotypes in Indian mustard germplasm described in the previous paragraph, a very different situation was revealed when a TuMV pathotype 8 isolate was inoculated to 55 breeding lines from an Indian mustard breeding program underway Australia, because most of them developed fully susceptible genotype + [204]. In such breeding programs, incorporation of resistance gene TuRBJu01 would offer valuable protection against most TuMV pathotypes found so far in Australia. This is because, unlike localized hypersensitive resistance (LHR = phenotype R_N_) which protects crops at the individual plant level, systemic hypersensitive resistance (SHR = phenotype +N_D_) protects them at the plant population level. It does this by killing infected plants, thus removing them as a source of infection for spread to neighboring plants [215]. When systemic hypersensitive resistance (= SHR, phenotype +_ND_) elicited by TuMV in presence of Indian mustard gene *TuRBJu01* was investigated by light microscopy and histochemical analysis of stem cross sections, it was associated with phloem necrosis, xylem occlusion, lignification, and hydrogen peroxide accumulation [215]. Phloem necrosis and xylem occlusion acted as the primary cause and secondary causes of SHR, respectively. When early TuMV infection before appearance of phenotype R symptoms (chlorotic spot local lesions) was investigated in Ethiopian mustard TZ-SMN using light microscopy and electrolyte leakage, it was shown to have initiated morphological changes associated with apoptotic-like programmed cell death and necrosis-like programmed cell death. Their development depended upon TuMV pathotype and the stage of infection reached [271]. Similar studies to these are lacking with LHR (phenotype R_N_).

Three genomes termed A, B and C occur alone or in different combinations in *Brassica* spp., B in black mustard, A and B in Indian mustard, A and C in canola, B and C in Ethiopian mustard [272]. In UK studies, dominant resistance genes *TuRB01, TuRB03, TuRB04* and *TuRB05* in canola, and *conTR01* in Chinese cabbage (*B. rapa*) were all found on the A genome. Recessive TuMV resistance gene (*retr01*) in Chinese cabbage was also on this genome. By contrast, quantitative trait locus *TuRB02* was on the C genome of canola [268,269,270,273,274]. Possibly, *TuRBJu01* is present on the A genome of Indian mustard and is related to canola genes *TuRB01* or *TuRB03* [202,208,264]. However, any resistance genes found responsible for the resistance phenotypes in back mustard and Ethiopian mustard in the future cannot be on the A genome as it is absent from them.

In Australia, control measures previously demonstrated to be effective at reducing virus spread in an analogous pathosystem involving a non-persistently aphid-borne virus are available for use with TuMV. They include: (i) deploying non-host barrier crops, (ii) promoting early crop canopy development and high plant density, reduce aphid landing rates, (iii) sowing into standing stubble, and (iv) avoiding or eliminating potential virus reservoirs with herbicide (see Table 1 for explanation of how these control measures operate). They were demonstrated previously in Australia with the bean yellow mosaic virus (BYMV; genus *Potyvirus*, family *Potyviridae*–narrow-leafed lupin pathosystem [33]. Plant breeding to enhance the TuMV resistance of future Australian canola and Indian mustard cultivars would require incorporation of additional resistance genes so they are protected against as many TuMV pathotypes as possible. With canola, suitable combinations of TuMV resistance genes that confer resistance to all TuMV pathotypes, apart from for the resistance-breaking pathotype, are already present in Australia. However, a major focus is required to search canola germplasm for sources of resistance to the resistance-breaking pathotype so that this too can be incorporated through breeding. With Indian mustard, as mentioned above, incorporation of resistance gene *TuRBJu01* would provide resistance against most TuMV pathotypes known to be in Australia, but a search of germplasm is needed to locate resistance to TuMV pathotype 3 and improved resistance to the canola resistance-breaking pathotype. A strategy to slow spread of the latter should include (i) area wide removal with herbicide of alternative volunteer crop and weed hosts that enable it to survive outside the annual growing oilseed growing season, and (ii) preventing movement of infected crop produce outside currently affected areas [210].

### 4.3. Tobacco Streak Virus

Tobacco streak virus (TSV; genus *Ilarvirus,* family *Bromoviridae*) was first reported in Australia in 1971 infecting dahlia and several weed species in south-east QLD [275]. Later in the 1970s, it was found in tobacco (*Nicotiana tabacum*) and strawberry (*Fragaria x ananassa*) in the same region [276,277]. TSV was also found in QLD or VIC infecting 11 weed species belonging to five different families (*Apocynaceae*, *Asclepiadaceae*, *Asteraceae*, *Lamiaceae, Solanaceae*) [13], and shown to be seed-transmitted in tomato and transmitted by *Thrips tabaci* (onion thrips) carrying TSV-infected pollen [278,279]. In the 1990s, a TSV outbreak in tobacco in QLD was attributed to its spread by the thrips species *Microcephalothrips abdominalis* (composite thrips) carrying TSV-infected pollen to the crop from the weed *Ageratum houstonianum* (blue billygoat weed) [280]. Success of TSV transmission by thrips required presence of highly TSV-infected pollen and release of TSV from this pollen near thrips feeding wounds [281,282].

Following more detailed studies involving serology, host range and transmission by different thrips species, three strains of TSV were described, TSV-Ag, TSV-S and TSV-A [275,277,283,284]. TSV-Ag was isolated from *A. houstonianum* and, based on serology, it was assumed synonymous with an earlier described strain TSV-As [280]. TSV-S was isolated from a clone of strawberry imported from North America [277,283,284], and TSV-A from the ornamental *Ajuga reptans* [285]. More recently, the characterization of complete (TSV-Ag and TSV-S) or partial (TSV-A) genomes obtained from archived reference samples revealed that all three ‘TSV strains’ are actually distinct virus species [286]. TSV-Ag is a species with proposed name ageratum latent virus (AgLV), TSV-S was shown to belong to strawberry necrotic shock virus (SNSV), and TSV-A was found to belong to tobacco streak virus but with evidence of recombination events. No further research on TSV was done between 1996 and 2004 in Australia, but this situation changed when it was identified as the cause of a devastating necrotic syndrome that caused major losses in the oilseed crop sunflower in QLD’s central highlands where it is sown widely [217,218,287].

In sunflower, the foliar disease symptoms TSV consist of necrosis (black streaking) of the leaf lamina, petiole, stem, and flower calyx, shortened internodes, weakened stems (Figure 6C), lodging of taller plants, and plant dwarfing (Table 2). When the plants of a susceptible sunflower hybrid are infected at an early growth stage, they are almost always killed (Figure 6D,E). Later infections often result in smaller, deformed seed heads and reduced seed quality [218,287]. After the early 2000s, TSV caused an overall yield loss of 20% in central QLD sunflower crops with >40% losses in many crops, and up to 70% losses in severely diseased crops, causing farmers to lose confidence in growing sunflower [217,218].

In central QLD, there are two biologically and genetically distinct TSV strains, TSV-parthenium and TSV-crownbeard, which have *Parthenium hysterophorus* (parthenium) and *Verbesina encelioides* (crownbeard) as their main weed hosts, respectively [219]. Both strains are transmitted readily in TSV-infected pollen carried by the thrips species *F. schultzei*, *M. abdominalis* and *T. tabaci, T. tabaci* being the least important vector in this region. The TSV-parthenium strain was seed-transmitted at a rate of up to 48% in naturally infected parthenium [217], and the TSV-crownbeard strain was seed-transmitted at a rate of up to 50% in naturally infected crownbeard [219]. Both TSV strains were readily seed-transmitted in experimentally infected *A. houstonianum,* with rates of up to 40% and 27% being recorded with TSV-parthenium and TSV-crownbeard, respectively [219]. By contrast, the two strains differ in *V. encelioides* as, although it was a natural host of TSV-parthenium, its infection resulted in non-viable seed. They also differed in *P. hysterophorus* as this was a poor host of TSV-crownbeard [219]. These differences act as a biological barrier that maintains their separation as both occur as distinct TSV strains despite often occurring in close proximity and being transmitted by the same vector species. There was evidence of possible RNA reassortment between them, but further studies are required to determine if the natural hybrids between them can persist [219]. In addition, in central QLD, the TSV-parthenium strain had a wider natural host range and occurred over a much greater geographical range. Moreover, in commercial sunflower crops TSV-crownbeard infections occurred far less often and caused less severe symptoms than TSV-parthenium infections. TSV-crownbeard’s occurrence was limited to the more restricted geographical distribution of *V. encelioides*. For these reasons and because it produces large amounts of pollen that are easily transported by thrips when epidemics occur in sunflower crops, TSV-parthenium is the predominant causal agent in central QLD [219].

Although it was not seed-borne in sunflower, in addition to being seed-borne in *P. hysterophorus* and *V. encelioides,* TSV is also seed-borne in other *Asteraceae* weed hosts, such as *Bidens pilosa* (cobblers peg) and *Conyza* sp. (fleabane) [217,219]. In addition, TSV-infected weed seeds become dispersed over distance by machinery, livestock, soil or harvested produce, thereby enabling establishment of the virus in new locations [217]. The virus also infects the grain legume crops chickpea and mungbean, but these are considered insignificant sources of infection for sunflower crops [218,219,220].

The summer period is when most of the annual rainfall occurs in central QLD [217]. Rainfed sunflower crops are planted in late summer to early autumn (February to March) which often coincides with the peak growth and flowering period of *P. hysterophorus* [220]. Planting sunflower under irrigation in spring (September to November), when there is much less *P. hysterophorus* flowering, greatly diminishes the risk of a severe TSV epidemic. The main drivers of TSV epidemics in sunflower crops in central QLD were identified as: (i) high populations of vector thrips carrying large amounts of infective pollen from external TSV host reservoirs into sunflower crops during warmer months of the year; (ii) the close proximity of large populations of TSV-infected *P. hysterophorus* to crops and their relative positions in relation to prevailing winds; and (iii) the absence of heavy regular rainfall periods in the critical growing season months of March and April as heavy rains diminished thrips populations [217,220].

When multiple field experiments were undertaken with TSV-parthenium and 23 sunflower hybrids at two sites in 2008 to 2012, useful partial resistance (i.e., far fewer plants became infected) or tolerance (i.e., where infected plants only developed mild symptoms) was identified in 10 hybrids, and glasshouse tests found that both also operated against TSV-crownbeard [220]. Apart from sowing sunflower hybrids with partial resistance or tolerance to TSV, other control recommendations arising from this study were: (i) using herbicide to remove *P. hysterophorus* and other weeds from within and around fields before sowing sunflower; (ii) sowing the crop upwind of any flowering *P. hysterophorus:* (iii) sowing a tall non-host barrier crop (sorghum) to diminish the risk of severe damage near to the crop edge; and (iv), when irrigation is available, manipulate sowing date to avoid planting in February to March [220].

In addition to being grown in central QLD, commercial sunflower oilseed crops are also grown on a large scale in subtropical southern QLD and northern NSW. They are also grown on a smaller scale in tropical north-west Australia, and over summer in the south-west Australian grainbelt [288]. Disease surveys of sunflower crops over several growing seasons in the late 2000s in southern QLD and northern NSW indicated that disease caused by TSV was absent in these regions (M. Sharman, unpublished data). TSV also causes some necrotic symptoms in three other Australian oilseed crops, cotton, safflower and soybean. This disease is of minor importance in cotton [218,219]. Also, although TSV causes disease in soybean, this crop is uncommon in the regions most affected by TSV [219]. The extent of its occurrence in commercial crops of safflower remains to be studied in Australia.

### 4.4. Cotton Bunchy Top Virus

In Australia, 80% of the cotton crop is irrigated but the remainder is semi-irrigated or rainfed. Cotton is grown under a subtropical climate in southern and central QLD and northern NSW, and under a warm temperate climate in central and southern NSW. In these regions, it is planted in spring, grown over summer and harvested in autumn [289]. In tropical northern Australia, it is grown in the cooler dry season [49]. Cotton bunchy top disease (CBTD) was first reported in Australia in the 1998–1999 growing season when devastating epidemics developed in cotton crops growing in four regions in NSW and QLD (Table 2). Up to 21% of cotton fields were badly affected by CBTD causing an estimated AUD$70 million loss [147,221]. CBTD was also reported from all other major cotton growing regions of QLD and NSW [222,223]. CBTD spread in cotton crops started at field edges or from isolated internal disease foci and was associated with *A. gossypii* (cotton aphid) which also transmitted it in glasshouse tests. It was not seed-borne. Outside the cotton growing season, it persisted in ratooned or volunteer cotton and its *A. gossypii* vector in several broad-leafed weed species. Almost all Australia cotton cultivars were CBTD-susceptible [221,222,290]. Although there were significant CBTD epidemics in central and southern QLD in early 2011, in general, serious epidemics have been uncommon. This was because of droughts that greatly diminished aphid vector numbers, and large-scale application of neonicotinoid seed treatments and foliar insecticides had the same effect within the crop. In the future, resistance to neonicotinoid insecticides in *A. gossypii* may cause CBTD epidemic reoccurrence [291]. Limited scale surveys for CBTD in cotton crops in tropical northern Australia did not find it (M. Sharman unpublished data), but continued surveillance for CBTD is required in these emerging cotton growing regions.

CBTD’s foliar symptoms depend upon the plant growth stage at time of initial infection and include pale light-green angular patterns on leaf margins (Figure 6F), downward cupping, marginal curling, deformation, reduction in size (Figure 6G), a leathery texture to mature leaves, and shortened petioles and internodes (Figure 6H). Late infection still causes obvious symptoms [147,222,223,290]. In glasshouse experiments, CBTD was associated with diminished photosynthetic rate, leaf area, plant height, number of bolls, and dry weight of bolls, roots and stem. Cotton lint yields were deceased by 67%, but no seed yield data were collected [222].

Early attempts to isolate and identify the graft-transmissible causal agent of CBTD failed, but it was considered likely to be an unknown member of the *Luteoviridae* [223]. Observations of CBTD suggested similarities to cotton blue disease which causes considerable damage to cotton crops in the Americas, Africa and Asia, and is caused by cotton leafroll dwarf virus (CLRDV; genus *Polerovirus*, family *Solemoviridae*). CBTD resembled cotton blue disease in foliage symptoms, and by being graft-transmissible and vectored by *A. gossypii* [222]. Moreover, cotton cultivars with a dominant resistance gene to cotton blue disease were also resistant to CBTD [147]. After a delay of more than a decade CBTD’s causal agent was finally identified. This was achieved when the partial genomic sequence obtained from CBTD-affected cotton leaf tissue was shown to resemble the sequence of CLRDV, but to be sufficiently different to be considered a new *Polerovirus* and given the name CBTV [147]. Two biologically and genetically distinct strains of CBTV have now been characterized in Australia, called CBTV-1 and CBTV-2. The partial genome reported by Ellis et al. [147] was for CBTV-1 but only CBTV-2 is closely associated with CBTD symptoms [224].

After CBTD’s causal agent was found, 15 plant species from five families were identified as natural or experimental hosts of CBTV in Australia [292]. The most frequently found CBTV hosts within and near to some cotton growing areas were volunteer or ratoon cotton and *M. parviflora*. Roadside volunteers growing ‘off-farm’ and more than one growing season old were often CBTV-infected suggesting they constitute an important threat as a long-term reservoir of this virus and of its *A. gossypii* vector, which can move it into cotton crops or cotton cropping areas. There were 11 CBTV host species in the *Malvaceae*, several of which are commonly found weeds in cotton growing areas. Although these are the only currently known alternative CBTV hosts, the virus probably has a wider natural host range that includes more non-*Malvaceae* species. In addition to ratooned and volunteer cotton plants, some of these weeds likely play important roles in CBTV’s survival outside the growing season.

After transgenic *Bt*-cotton was introduced into Australia to control *Helicoverpa* spp. (cotton bollworm) and other pests in the mid-1990s, the reduction in foliar insecticide applications that followed and the inactivity of *Bt* towards *A. gossypii,* allowed aphid population numbers to increase [291]. Nevertheless, as mentioned earlier in this Section, large-scale application of neonicotinoid seed treatments and other foliar insecticides to cotton still suppressed *A. gossypii* populations well, and this approach was effective at minimizing CBTV spread. However, due to (i) the recent development of neonicotinoid insecticide resistance in *A. gossypii,* (ii) the likelihood of its withdrawal for use in Australia in the future, and (iii) the susceptibility of Australian cotton cultivars to CBTV, there is a need explore the effectiveness of other types of insecticides with different chemistry, and phytosanitary, cultural and host resistance control measures, to control CBTV. Herron and Wilson [293] recently described possible measures for controlling *A. gossypii* as they relate to the risk of CBTV spread and encouraged a precautionary approach to manage the risks arising from CBTV infection and insecticide resistance.

In the future, sustainable control of CBTD in commercial cotton crops may be underpinned by incorporating the recently characterized genetic resistance to it conferred by the putative single dominant locus *Cbt* into new cotton cultivars [294]. The *Cbt* locus was found to be located on chromosome A10 close to resistance gene *Cbd* to CLRDV mapped by Fang et al. [295]. Ellis et al. [294] noted that while their mapping indicated that *Cbt* was close to, but distinct from, *Cbd,* they could not rule out the possibility that the same gene is responsible for resistance to both viruses.

### 4.5. Other Oilseed Viruses

Cauliflower mosaic virus (CaMV; genus *Caulimovirus,* family *Caulimoviridae*,) and broccoli necrotic yellows virus (BNYV; genus *Cytorhabdovirus*, family *Rhabdoviridae*) both infect canola in Australia (Table 3) [13]. CaMV occurs in TAS, NSW, VIC, SA and WA [13,199,200,244,296]. It is vectored by aphids in a semi-persistent manner but is not seed-borne. In Australia, *M. persicae* and *B. brassicae* are reported as its main vectors, although other species are likely to be involved [13,296]. In 1998–1999 surveys of canola in south-west Australia, CaMV was detected in 27% and 2% of crops in 1998 and 1999, respectively. CaMV incidences in infected crops were 1–17% (1998) and 1% (1999), six of 10 cultivars were infected and it occurred in low, medium and high grainbelt rainfall zones. Chlorotic ringspot symptoms were associated with CaMV infection in canola and the weed *R. raphanistrum* was identified as a significant infection reservoir for its spread to crops [199]. In surveys of spring-sown dual-purpose canola growing in 2016 in the high rainfall zone of southern Victoria, 83% of canola crops surveyed were CaMV infected, the average crop incidence was 8% and infection was found in up to 39% of plants [244]. Its recorded incidences in canola crops mostly seem insufficient to cause economic losses in canola. However, it has the potential to cause losses in individual canola crops in years that favor widespread infection [262]. In 2004, CaMV infection was reported to occur commonly in Indian mustard crops and *Brassicaceae* weeds in northern NSW [200], but no incidence data are available for these hosts. BNYV is reported from SA, TAS and VIC and is transmitted by the aphid vector *B. brassicae,* but is not considered of any economic importance [13,297]. CaMV and BNYV sometimes occur in mixed infection [13]. An unidentified virus (or viruses) belonging the *Mastrevirus* genus (family *Geminiviridae*) was detected infecting occasional plants of canola and Indian mustard in northern NSW, but its incidence was too low to be of economic importance [298].

‘Sunflower ringspot virus (SRSV)’ was described infecting sunflower in southern QLD and was originally thought to be an *Ilarvirus* [299,300]. Its foliage symptoms in sunflower include bright chlorotic rings, mosaic and line patterns. However, recent high throughput sequencing of an ‘SRSV’ isolate collected in 2011 identified it as pelargonium zonate spot virus (PZSV; genus *Anulavirus,* family *Bromoviridae*) (Table 3). Moreover, when PZSV-specific RT-PCR tests and sequencing of partial coat protein genes were done on a further five sunflower or tomato ‘SRSV’ isolates archived from as early as the mid-1970s and originally from southern QLD or northern NSW, PZSV was the only virus present (M. Sharman, unpublished data). Therefore, all previous records of ‘SRSV’ from Australia appear to be PZSV. Disease symptoms due to PZSV infection occur infrequently in sunflower crops in northern NSW and southern QLD. PZSV has also been detected infecting volunteer canola in southern QLD (M. Sharman, unpublished data). While seed transmission of PZSV has been reported in tomato [301], similar studies are needed to test if seed transmission occurs in other crops including sunflower. It can also infect the *Brassicaceae* [302], but there have been no studies examining its occurrence in Australian brassica oilseeds crops. PZSV was first reported in Australia in 2010 in WA infecting the introduced weed *Cakile maritima* (European searocket) and the Australian native plant species *Anthocercis ilicifolia* [303,304]. In earlier studies, cucumber mosaic virus (genus *Cucumovirus*, family *Bromovirideae*) and watermelon mosaic virus (genus *Potyvirus*, family *Potyvirideae*) were reported infecting the oilseed crops sunflower and sesame, respectively, both in QLD [13]. However, these appear isolated occurrences.

Several additional viruses were reported infecting Australian soybean crops more than 30 years ago (Table 3). Amongst them, soybean mosaic virus was found infecting soybean in QLD and NSW. The others were all found infecting soybean in QLD. They included alfalfa mosaic virus (genus *Alfamovirus*, family *Bromovirideae*), glycine mosaic virus (genus, *Comovirus*, family *Secovirideae*), lucerne Australian latent virus (genus, *Nepovirus*, family *Secovirideae*), passionfruit woodiness virus (PWV, genus *Potyvirus*, family *Potyvirideae*) and peanut mottle virus (PeMV; genus *Potyvirus*, family *Potyvirideae*) [13]. In 2016, cowpea mild mottle virus (genus *Carlavirus,* family *Betaflexiviridae*) was detected in QLD infecting a soybean crop with foliage symptoms of leaf distortion and mottle occurring at a disease incidence of 8%. It was transmitted by *Bemisia tabaci* (silver leaf whitefly) [305]. Currently, none of these viruses constitute a serious threat to Australian soybean oilseed production. However, in 2016 soybeans growing in parts of southern QLD were severely impacted by a virus-like disease caused by phytoplasma, resulting in almost 100% yield losses [306]. Symptoms in soybean were variable depending upon time of infection, and included severe phyllody, tiny sterile pods, or more mild thickening, yellowing and curling of upper leaves [307].

Australian peanut crops growing in QLD sometimes become infected with tomato spotted wilt virus (TSWV) and capsicum chlorosis virus (CaCV) (both genus *Orthotospovirus*, family *Tospoviridae*,), and by PeMV and PWV (Table 3) [308,309]. CaCV causes a less common and severe necrosis disease than TSWV in affected peanut crops and infections with it are usually found in close proximity to the major alternative host of CaCV, *Ageratum conyzoides* [310]. Although currently these four viruses rarely cause significant economic losses in peanut oilseed production in Australia, TSWV causes enormous losses in peanut crops elsewhere, e.g., to the south eastern USA’s peanut industry [311] so it still poses a significant future threat. Recently, outbreaks of virus-like disease in peanut crops caused by phytoplasma occurred in QLD. The disease symptoms in peanut closely resemble peanut kernel shrivel (PKS) syndrome, a disorder estimated to cause AUD$1 million of damage in some growing seasons. This has reduced farmer confidence in growing peanuts and limited their production [306]. The brown leafhopper (*Orosius orientalis*) was an experimental vector (M. Sharman, unpublished data), but other leafhopper species might be involved in these virus-like disease epidemics.

### 4.6. Recommendations for Further Research

More research is required to better understand the biological differences between genetically diverse TuYV isolates, and TuYV’s epidemiology particularly the drivers behind autumn aphid vector activity. In addition, field experimentation is needed to establish whether (i) insecticides other than neonicotinoids and (ii) cultural control measures are effective at reducing TuVY spread. Australian canola breeding should prioritize developing new cultivars with TuYV resistance. Testing of flying *M. persicae* caught in strategically located traps should be continued as an early warning system for TuYV epidemics and should be used to re-calibrate the forecasting model of Maling et al. [235] to take into account current cultural practices and climate alterations so it can be used as a decision support system tool. This model should also be calibrated to accommodate current conditions in south-eastern Australia.

Large-sale surveys are required to establish the current incidence of the TuMV resistance breaking strain across Australian canola producing regions. Also, a large-scale screening program with canola germplasm is needed to identify sources of resistance to this strain suitable for resistance breeding. In addition, more research is required on TuMV’s epidemiology, to quantify the yield losses it causes in Australian canola and Indian mustard crops, and the extent to which cultural control measures are able to diminish its spread. Research is also required to establish which additional virus resistance genes other than *TuRBJu01* are linked to the different resistance phenotypes found in Indian mustard germplasm so they can be incorporated into new cultivars carrying comprehensive resistance, including to the canola resistance-breaking strain.

Research is needed to understand the nature and genetic control of the resistance and tolerance to TSV found in some sunflower cultivars. Also, more research is required on TSV’s epidemiology in the presence or absence of irrigation in different sunflower production systems. An integrated disease management approach is required for control of TSV in sunflower crops. To achieve this, all new Australia sunflower cultivars need to be screened for resistance and tolerance to TSV before release. Also, additional research is needed to increase the effectiveness at decreasing TSV spread of: (i) removing TSV weed hosts using herbicides or mechanical means before planting; and (ii) modifying agronomic practices to deter thrips landings. Ideally, the ultimate aim should be to establish a forecasting model and decision support systems to alert farmers over the need for control measures both before sowing and subsequently during crop growth.

To reduce the need for aphid vector control using insecticides, and the risk of developing further insecticide resistance, a better understanding of CBTD epidemiology is necessary. Knowledge gaps that require attention include establishing: (i) which weed hosts play critical roles as virus and *A. gossypii* reservoirs; (ii) the spatial dynamics of CBTV spread; (iii) the annual patterns of aphid vector activity in relation to weather variables; and (iv) the seed yield losses CBTV causes in cotton. Further work is also required to search for CBTV resistance-breaking strains and investigate if natural recombinants of CBTV-1 between CBTV-2 have different disease or host infection characteristics to those of either parental virus. Continued monitoring of emerging cotton production regions in tropical northern Australia establish if CBTV, or other virus threats, are causing production losses is also warranted.

Since there is no information over the virus diseases that afflict linseed in Australia, this constitutes a knowledge gap needing to be addressed.

## 5. Future Threats to Virus Management

### 5.1. Biosecurity Threats

Australia has a considerable advantage over many other countries that enables it to impede introduction of plant virus diseases that would otherwise threaten the country’s cereal and oilseed industries. This advantage arises from having: (i) the presence of an agricultural system involving cultivation of native plants which used different crop species to the ones cultivated after European settlement in 1788; (ii) a maritime border that encircles the entire continent; and (iii) strict plant biosecurity regulations. In consequence, many virus diseases that severely damage cereal and oilseed crops in other parts of the world are still absent. There is, however, an ever-increasing risk of their introduction in planting material (=vegetative propagules or seeds). This is due to factors like: agricultural globalization; trade facilitation by increasingly rapid air and sea transportation methods; relaxation of plant quarantine regulations to conform with less rigorous World Trade Organization rules; agricultural intensification, diversification and expansion associated with agricultural development; and climate change-driven influences [5,6,7,8,9,53,312]. Avoiding the arrival of economically important cereal and oilseed viruses, and their vectors, requires biosecurity measures applied by both the exporting country (pre-border) and the importing country (border). In addition, preventing their establishment, or containing their spread, requires biosecurity measures after arrival (post-border) [7,8,15,312,313]. Australian import biosecurity conditions require a range of measures to be enacted in pre-border and border situations. These include pre-arrival inspection and plant health certification of planting material, and on-arrival inspection at airports and seaports followed by mandatory growth in a regulated PEQ facility where plants and seeds are evaluated for virus presence. This is done visually for virus symptoms and by active diagnostic testing for specific viral pathogens as required by Australian federal biosecurity guidelines. After importation requirements are satisfied, healthy planting material can be released [15,312,313,314]. Should attempts to prevent virus entry and subsequent establishment fail, prompt post border application of eradication or containment procedures is required to avoid further spread [7,8,15,312]. Similar considerations to those applying to virus disease threats also apply to the biosecurity measures associated with the pre-entry, pre-border, border and post-border situations that concern preventing the entry and subsequent establishment of their insect, mite, nematode or fungal vectors [312].

Another way viruses previously absent from Australia may arrive is via viruliferous insect vectors blown from neighboring Southeast Asian countries into northern Australia by monsoonal winds [315,316]. The same applies to the introduction of virus carrying insect vectors reaching south-east Australia (or TAS) on wind currents from New Zealand. In a similar way, insect vectors previously absent from Australia can arrive in northern or south-east Australia on wind currents from neighboring countries. Moreover, migrating birds carrying seed-borne viruses in their guts, or food debris containing viruses left behind by fishermen from nearby counties visiting Australia’s shores temporarily, can provide additional virus introduction avenues [317]. Addressing virus and insect vector entry by these means rather than through seaports or airports requires finding those that arrive by rigorous area-wide surveillance, as conducted by the Northern Australian Quarantine Strategy [318]. When found, as with viruses and vectors arriving via seaports and airports, prompt application of eradication or containment procedures is required to avoid further spread (post border) [7,8,15,312].

Examples of cereal or oilseed viruses and vectors that are thought to have become established in Australia within the last 40 years include: WSMV introduced in wheat seed from North America (see Section 3.2 above); *P. graminis* introduced in imported cereal seed lots contaminated with its resting spores (see Section 3.5 above); and the cereal aphids *M. dirhodum* (see Section 3.1 above) and *Diuraphis noxia* (the Russian wheat aphid) [319], both likely introduced inadvertently on imported plant material. *D. noxia* is a vector of brome mosaic virus (BrMV; genus *Bromovirus*, family *Bromoviridae*) [320], a seed-borne cereal virus not yet present in Australia. Examples of major plant virus diseases that occur elsewhere and therefore pose a biosecurity threat to Australian cereal and oilseed industries are in Table 4. For each causal virus or virus complex, Table 4 provides information on their crop hosts, vector(s), current global distribution and disease impact.

**Figure 7:**
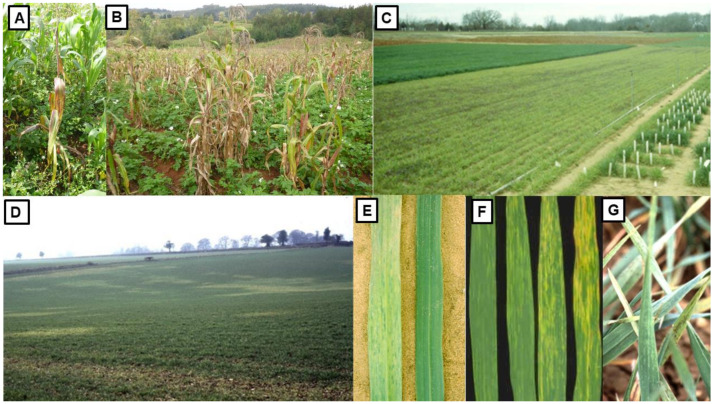
Examples cereal virus disease threats of concern for Australia. (**A**) Maize plant dying from maize lethal necrosis disease (center), surrounding maize plants healthy. (**B**) Smallholder maize crop in Kenya destroyed by maize lethal necrosis disease (all maize plants dying or killed), potato understory crop unaffected. (**C**) Wheat plots and rows exposed to soil-borne wheat mosaic virus (SBWMV) infection in the field in the USA: plants of susceptible cultivar (central plot) and breeding lines (bottom right rows) show leaf chlorosis and stunting whereas plants of resistant cultivar (top left plot) and breeding lines (bottom right rows) show heathy vigorous growth. (**D**) Barley field in the UK showing irregular shaped patches of chlorotic, dwarfed plants caused by infection with a mixture of barley yellow mosaic virus (BaYMV) and barley mild mosaic virus (image credit: Rothamsted Experimental Station/M. Adams). (**E**). Wheat leaf infected with SBWMV showing chlorotic leaf streaking (left) versus healthy leaf (right) (**F**) Wheat leaves infected with wheat spindle streak virus showing ‘spindle-shaped’ streaks. (**G**) Leaves of barley plants infected with BaYMV showing chlorotic streak and blotch symptoms (image credit: Rothamsted Experimental Station/M. Adams). Images modified: (**A**) from [53] (**B**) from [8]; (**C**,**E**,**F**) from [326]; (**D**,**G**) from [324]. Image credits (**C**,**E**) @Oklahoma State University/R.M. Hunger; (**D**,**G**) @ Rothamsted Experimental Station/M. Adams; (**F**) @University of Kentucky/Anonymous.

The virus disease of greatest concern for Australian maize and sweet corn production should it become established is maize lethal necrosis (MLND; Figure 7A,B) caused by a bi-partite virus complex (Table 4) [8,321]. The viruses involved in this complex consist of maize chlorotic mottle virus (MCMV; genus *Machlomovirus, genus, Tombusviridae*)*,* and a member of the *Potyviridae*, usually MDMV, SCMV or WSMV. MCMV, MDMV, SCMV and WSMV are all seed-borne in maize [8,103,321]. Moreover, two of these three *Potyviridae* are already widespread in Australia (SCMV and WSMV), as are their aphid (SCMV) and eriophyid mite (WSMV) vectors (see Section 3.2 and Section 3.3 above), and beetle and thrips species potentially capable of transmitting MCMV (Table 4). Although yet to be demonstrated, since JGMV is a potyvirus that is widespread in Australia (see Section 3.3 above), should MCMV become established it would likely interact in mixed infection with JGMV causing MLND in maize and sweet corn crops. In addition to infecting maize and sweet corn, MCMV, MDMV, SCMV and WSMV also cause diseases in other cereal crops when present in single infections, including barley, pearl millet, sorghum and wheat (MCMV), oats, pearl millet and sorghum (MDMV), sorghum (SCMV), and barley and wheat (WSMV) [103]. The most damaging of these virus-cereal pathosystems include WSMV in wheat which already occurs in Australia (see Section 3.2 above), and MDMV in maize, sweet corn and sorghum [322], both of which pathosystems are not yet found in the country. Moreover, as MDMV causes the most important and damaging virus disease of sorghum worldwide [322], its establishment would be cause for particular concern for this crop. In addition, the establishment within Australia of maize rough dwarf virus (MRDV, genus *Fijivirus*, family *Reoviridae*) and maize streak mosaic virus (MSMV; genus *Mastrevirus*, family *Geminiviridae*) would also be of considerable concern for Australian maize and sweet corn production since both cause major losses in these crops in other parts of the world (Table 4) [7]. Three devastating virus diseases that would pose a major threat to rice production should they become established in Australia are rice tungro (tungro = ‘degenerated growth’ in Filipino), rice hoja blanca (hoja blanca = ‘white leaf’ in Spanish) and rice yellow mottle. Rice tungro disease is caused by a bi-partite complex of rice tungro baciliform virus (RTBV; genus *Tungrovirus*, family *Caulimoviridae*) with rice tungro spherical virus (RTSV; genus *Waikavirus*, family *Secoviridae*). Rice hoja blanca disease is caused by rice hoja blanca virus (RHBV; genus *Tenuivirus*, family *Bunyaviridae*), and rice yellow mottle disease by rice yellow mottle virus (RYMV; genus *Sobemovirus*, family *Solemoviridae*) (Table 4) [7,8]. Triticum mosaic virus (TriMV; genus *Poacevirus*, family *Potyviridae*) causes substantial grain yield losses in wheat when it occurs in mixed infection with WSMV, which is often found in North America. The resulting disease is due to a synergistic interaction between these two viruses [323]. Since WSMV is already widespread (see Section 3.2 above), this would be likely to happen in Australia if TriMV became established.

A range of soil-borne cereal viruses are transmitted by the vector *P. graminis* which has motile zoospores that swim in soil moisture [324,325,326,327,328,329] and occurs in Australia (see Section 3.5 above). Their likely pathway for entry to Australia would be via imported cereal seed contaminated with viruliferous *P. graminis* resting spores, as was suggested previously for SBWMV when it reached New Zealand (see Section 3.5 above). For barley, wheat, pearl millet, maize, sorghum and rice growing under warm conditions, the viruses concerned are peanut stunt virus (PCV) and Indian peanut stunt virus (IPCV) both in genus *Pecluvirus* (family *Virgaviridae*) (Table 4). Both occur currently in high rainfall, subtropical and subtropical regions in Sub-Saharan Africa or the Indian subcontinent, respectively [325,326,327,328]. Therefore, once established in soil of infested fields, they would be likely to cause serious disease problems in cereal crops growing in warmer parts of Australia (NT, QLD and northern WA and NSW). By contrast, the other soil-borne cereal viruses are adapted to high rainfall regions in parts of the world where cereals are grown under cooler temperatures, such as parts of Europe, East Asia, and both North and South America (Table 3) [325,329]. In Australia, such climatic conditions occur during the winter growing season in the south-east, south, and south-west grainbelts, and TAS. For wheat, the viruses involved are SBWMV (Figure 7C,E), SBCMV, WSSMV (Figure 7F; Table 4), Chinese wheat mosaic virus (CWMV; genus *Furovirus*, family *Virgaviridae*), and wheat yellow mosaic virus (WYMV; genus *Bymovirus*, family *Potyviridae*) (Table 4). For barley, they are SBWMV, and the genus *Bymovirus* (family *Potyviridae*) viruses barley yellow mosaic virus (BaYMV) and barley mild mosaic virus (BaMMV) (Figure 7D,G; Table 4). For oats, they are oat mosaic virus (OMV; genus *Bymovirus,* family *Potyviridae*), and oat golden stripe virus (OGSV; genus *Furovirus*, family *Virgaviridae*), and, for triticale and rye, they are SBCMV, SBWMV and WSSMV (Table 4). *P. graminis* vectored viruses would be unlikely to arrive in Australia all at the same time but gradually over a longer period. However, once established, they would be very difficult to eradicate from infested field soils due the very long-term resilience of virus-contaminated *P. graminis* spores resting in the absence of host crops [324,325,326,327,328,329].

To date, the Australian cotton industry has avoided the damage to international cottonseed oil production from cotton blue disease caused by infection with CLRV (see Section 4.4 above), which currently occurs in Sub-Saharan Africa, Southeast Asia, and North and South America (Table 4). The same applies to cotton leaf crumple disease caused by cotton leaf crumple virus (CLCrV; genus *Begomovirus*, family *Geminiviridae*), present in the Indian subcontinent, and Central and North America, and cotton leaf curl disease caused by cotton leaf curl viruses (CLCuVs; genus *Begomovirus*, family *Geminiviridae*), present in the Indian subcontinent, East Asia, Southeast Asia, and North, East and West Africa (Table 4) [103,224,330]. However, CLRV’s aphid vector (*A. gossypii*) is very common in subtropical and tropical regions of Australia [291,293], as is the whitefly vector of CLCrV and CLCuVs (*B. tabaci*) in warmer regions of eastern Australia [331]. Therefore, should any of these viruses become established in cotton growing areas of QLD and NSW, and CLRV in such areas in the NT and WA, serious outbreaks would be expected. Indeed, CLRDV infects cotton in East Timor which is only a short distance from Australia’s northern coastline [332], and in Thailand [333]. This highlights the biosecurity threat this virus poses to emerging cotton production regions in northern Australia (Figure 1). Also, CLRDV is most likely synonymous with chickpea stunt disease associated virus (CpSDaV; genus species) which infects peanuts [334] so its arrival could have wider implications towards other oilseed crops.

Major biosecurity virus threats to Australian peanut oil production include damaging diseases caused by the *P. graminis* transmitted viruses PCV and IPCV (see above in this Section) (Table 4). They also include: groundnut rosette disease, which occurs in Sub-Saharan Africa and its offshore islands, and is caused by a tri-partite virus complex consisting of groundnut rosette virus (GRV; genus *Umbravirus*, family *Tombusviridae*), groundnut rosette assistor virus (GRAV; genus *Luteovirus*, family *Tombusviridae*) and a virus satellite; and groundnut bud necrosis disease which is present in the Indian subcontinent, Central and East Asia and Southeast Asia, and is caused by groundnut bud necrosis virus (GBNV; genus *Orthotospovirus*, family *Tospoviridae*) (Table 4) [7,103,335]. Both diseases would likely establish readily should their causal viruses become introduced. This is because the insect vector for GRV and GRAV is *A. craccivora,* which occurs commonly throughout Australia [13,107], and for GBNV its insect vectors are *Thrips palmi* (melon thrips) and *F. schultzei* (common blossom thrips). *T. palmi* has a widespread distribution across northern Australia, and *F. schultzei* occurs commonly throughout the Australian continent [335]. Groundnut bud necrosis disease also causes major losses in soybean production across the Indian subcontinent, Central and East Asia and Southeast Asia [7,335], so if it became established, Australian soybean oil production would likely be threatened. Several other virus diseases not listed in Table 4 afflict soybean, oilseed brassica and sunflower crops in other parts of the world [103,300,336,337], and some of these may become major biosecurity threats for Australia as circumstances change in the future.

Worldwide, PCR, tissue blot immunoassay (TBIA), ELISA and biological indexing were formerly the standard procedures for detection of cereal and oilseed viruses with PEQ. However, biological indexing is both labor-intensive and time consuming, and obtaining supplies of reliable and accurate antisera to support TBIA and ELISA poses a significant limitation to their use. In addition, they are sometimes unable to identify diverse virus variants. Fortunately, the cost of high throughput sequencing has declined rapidly due to advances in sequencing technology, so it is now feasible to use it to overcome such drawbacks and deploy as a routine diagnostic tool for detection of cereal and oilseed viruses in PEQ [338,339].

Future research efforts need to focus on developing more robust and cost-effective diagnostics and surveillance strategies to help avoid establishment of damaging cereal and oilseed viruses and their vectors within Australia. This includes improving diagnostic standards at the border and providing tools to support cost effective biosecurity measures both there and during area-wide surveillance. In addition, with appropriate modifications, such as the metabarcoding already used for insect identification [340], high thoughut sequencing could offer a paradigm shift in PEQ routine cereal and oilseed virus testing procedures.

### 5.2. Threats from Vector Insecticide Resistance

Although insecticides are ineffective at controlling non-persistently insect transmitted viruses [10], they can provide effective control of viruses transmitted persistently by insects, such as BYDV in cereals, TuYV in canola and CBTV in cotton (see Section 3 and Section 4 above). Australian cereal and oilseed farmers rely mostly on chemical control by insecticide applications to manage vectors of persistently insect-transmitted viruses, and, in consequence, the viruses they transmit. Prophylactic application of neonicotinoid (group 4A) seed treatments is common practice in Australian cereal and oilseed crops, such as wheat, barley, oats, canola and cotton, with many seed companies selling pre-treated seed. Such seed treatments have proven particularly effective at protecting Australian canola crops from infestation with *M. persicae* and infection with TuYV, resulting in increased yields (see Section 4.1 above). They also yield smaller benefits from reduced direct aphid feeding damage to Australian canola crops [341]. Treatment of cereal seed with neonicotinoid insecticides has also proven particularly effective at protecting wheat, barley and oat crops from yield losses to YDV’s (see Section 3.1. above). Alternatively, before seed dressings were used prophylactically, two foliar sprays of pyrethroid insecticide applied during the critical early cereal crop growth stage were used widely for BYDV control in Australia [70]. However, there are three major threats to this current paradigm: (i) biosecurity incursions of insect vector populations carrying new insecticide resistance traits; (ii) insecticide resistance development in local insect vector populations; and (iii) deregistration and banning of specific insecticide groups due to their off-target impact on other components of local ecosystems.

Several major aphid, thrips and whitefly vector species of important virus diseases of cereal and oilseed crops readily develop resistance to different classes of insecticides [252,253,254,291,293,335,342], and eriophyid mite vectors that transmit semi-persistently [153] have also proven impossible to manage using miticides [343]. The key major vectors species which have developed pesticide resistance mechanisms include ones that transmit viruses of current, or future, concern for Australia. These are the aphid species *A. gossypii* (CBTV and CLRDV vector) and *M. persicae* (TuYV vector), the whitefly species *B. tabaci* (CLCuV and CLCrV vector), the thrips species *Frankliniela oxidentalis* (TSWV vector) and *Thrips palmi* (GBNV vector), and the eriophyid mite vector WCM (WSMV vector) (Table 2, Table 3 and Table 4). This makes effective long-term chemical control of these vectors and any viruses they transmit reliant upon the development of chemicals with new modes of action and the adoption of strict insecticide application regimes involving non-prophylactic use and rotation between different insecticide classes.

In addition to behavioral resistance involving insecticide avoidance, e.g., in eriophyid mites [343], three different insecticide resistance mechanism classes occur in insects: (i) target site resistance conferred by point mutations in insecticide target sites linked to insect nervous systems which substantially reduce insecticide efficacy; (ii) metabolic resistance conferred by enhanced expression of genes that encode detoxification enzymes the magnitude of which dictates the level of resistance; and (iii) reduced penetration or increased excretion of insecticide [344,345]. Several different resistance mechanisms have been identified in important insect vectors most of which already occur in Australia. These include: (i) knockdown resistance (*kdr*-type) conferred by multiple point mutations in voltage-gated Na+ channels effective against pyrethroids and the now banned organochlorines; (ii) modified acetylcholinesterases (MACE) conferred by mutations at the AChE target site effective against carbamates; (iii) insensitive gamma-aminobutyric acid (GABA) receptors effective against cyclodienes and phenylpyrazoles; (iv) over-production of esterases E4 or FE4 effective against organophosphates, carbamates and, to a lesser extent, pyrethroids; and (v) over-production of cytochrome P450 genes reducing sensitivity to neonicotinoids [344].

Australian populations of *M. persicae* have developed target site resistance to carbamates (S431F mutation) and synthetic pyrethroids (L1014F, M918T and M918L mutations), strong metabolic resistance to organophosphates and carbamates (enhanced expression of E4/FE4 esterase), and mild metabolic resistance to neonicotinoids (enhanced expression of cytochrome P450 gene CYP6CY3) [252,253,254,346]. Furthermore, probably due to over-reliance over several years as the most reliable foliar insecticide option, there is now evidence of reduced sensitivity to sulfoxaflor in *M. persicae* in Australia, although the resistance mechanism involved has not yet been elucidated [254]. Although it has developed in other world regions, target site resistance to neonicotinoids conferred by the R81T mutation in the nicotinic acetylcholine receptor (nAChR) beta subunit which also confers some cross resistance to sulfoxaflor is yet to be detected in Australian *M. persicae* populations [347,348]. Australian *A. gossypii* populations have also developed resistance to carbamates and organophosphates (S431F, S302F and A302S mutations in the AChE gene Ace1), pyrethroids (L1041F mutation) and metabolic resistance to neonicotinoids (enhanced expression of a cytochrome P450 gene) [291,293,342,349]. Although there are no reports of Australian populations of *A. gossypii* having developed resistance to sulfoximines or the butenolide insecticide flupyradifurone (group 4D) yet [349], metabolic resistance to these compounds conferred by overexpression of cytochrome P450 genes is present in other parts of the world, such as China [350]. Furthermore, *A. gossypii* has also been reported to develop target-site resistance to neonicotinoids (R81T mutation) [351]. Resistance to insecticides in Australian populations of *B. tabaci* MEAM1 (=virus vector bioytpe B) is reported against organophosphates, carbamates, synthetic pyrethroids and the insect growth regulator pyriproxyfen, but not yet against neonicotinoids [352]. Outside Australia, *B. tabaci* MEAM1 populations have developed resistance to neonicotinoids such as imidacloprid [353], but apparently not yet to sulfoximines or flupyradifurone [354,355]. Therefore, the possibility of *M. persicae* and *A. gossypii* populations with stronger resistance to neonicotinoids and sulfoxamines, and *B. tabaci* MEAM1 populations with neonicotinoid resistance arriving in imports, presents three serious vector biosecurity threats for Australia. They pose a threat to Australian canola and cotton production because both industries rely heavily on these two insecticide groups to control insect vectors. Uncontrolled populations of virus vectors in these crops would lead to increased virus epidemics and resulting losses from TuYV in canola and CBTV in cotton. Such uncontrolled vector populations would also increase damaging epidemics of CLRDV, CLCuSV and CLCrV in cotton should these viruses become established in Australia. They would also increase the insect vector reservoir for viruses of other crops. As in other parts of the world, in Australia *F. occidentalis* can no longer be controlled by any of the major groups of insecticides, only by newer groups such as the spinosyn insecticides spinosad and spinetoram [356]. Similarly, Australian populations of *T. palmi* also cannot be controlled by applying any of the major insecticide groups, application of insecticidal soaps constituting the only current recommendation for their management using chemicals [357].

Although local clones of some populations of Australian *R. padi*, the primary vector of BYDV and CYDV in cereals, have developed tolerance to the pyrethroid alpha-cypermethrin, this aphid vector species remains susceptible to most other insecticides used to control it in Australia [358]. However, in other world regions, including East Asia and Europe, it has developed resistance mechanisms to carbamates, organophosphates and neonicotinoids [125,359,360,361]. Furthermore, *S. avenae*, a YDV vector not yet found in Australia, has developed a broad insecticide resistance profile [125,126] (see Section 3.1 above). Therefore, the possibility of arrival of *R. padi* or *S. avenae* populations with multiple insecticide resistances represents two further vector biosecurity threats for Australia.

Many studies have shown that widespread and prophylactic use of non-specific industrial insecticides, such as currently employed by the Australian grains industry, often has negative impacts on other animals in the environment which disrupts ecosystem stability. Unfortunately, this also includes negative impacts on beneficial insects such as pollinators and insect predators or parasites that are allies in controlling crop insect pests. For example, neonicotinoids have lethal or sub-lethal impacts on beneficial insects and have been associated with a >75% decline in total insect flying biomass over a 27-year period [362,363]. Neonicotinoids also negatively impact other fauna including birds, bats and aquatic organisms [364]. This knowledge concerning the unintended consequences of widespread neonicotinoid use has greatly increased public scrutiny. This has led to their banning in Europe; in 2013 for use with canola and in 2018 with all field crops. Other similar chemistries, such as sulfoxaflor and flupyradifurone, are also under scrutiny following recent studies showing negative environmental impacts e.g., [365,366]. Unfortunately, the banning of neonicotinoids had immediate negative economic implications, including for cereal and canola production, which is forcing agricultural industries to seek emergency use authorizations or explore environmentally friendly alternatives [367,368]. Deregistration and banning of key insecticide groups may also favor insecticide resistance development as it increases reliance on the reduced number of available compounds remaining, thus increasing selection pressure for, and the likelihood that, they will develop insecticide resistance [361]. 

Bans on certain insecticide groups seem likely to occur in Australia in the near future, so our cereal and oilseed industries must be proactive about preparing for life without them. This necessitates investment in research and development of new non-insecticidal control measures that suppress the spread and impact of virus diseases, such as new technology to generate durable broad-spectrum host resistance (e.g., CRISPR-cas system), biopesticides, RNAi and nanoparticles [64,65,369]. Furthermore, continued research, extension and adoption of existing cultural and phytosanitary control measures should still be undertaken. In addition, if utilized non-prophylactically and proactively, an insecticide resistance management approach employing some synthetic insecticides would still have a role to play in future integrated disease management of persistently insect-transmitted virus diseases of cereals and oilseed crops in Australia. Ultimately, a robust integrated management approach incorporating multiple sustainable control measures that promote a healthy ecosystem containing abundant natural predators and parasites of vectors should be the long-term goal.

### 5.3. Threats from Resistance Breaking Virus Strains

The likelihood that resistance-breaking virus strains will develop constitutes a major hazard when single gene virus resistances are being incorporated into new crop cultivars by plant breeding programs. It also applies when new cultivars are unknowingly bred from parental lines with resistance genes, as with TuMV in canola [210]. In the past, widespread infection with TuMV in canola was limited due to presence of one or more TuMV resistance genes amongst almost all the canola cultivars grown in Australia (see Section 4.2 above). This protection still occurred despite the widespread occurrence of TuMV infection in weeds that act as a reservoir of infection for its spread to crops, especially in NSW. However, the situation recently changed with the appearance and spread of a resistance breaking TuMV strain that overcomes all the TuMV resistance genes present in these cultivars (see Section 4.2 above). Similarly, over the last two decades widespread infection with JGMV in maize and sorghum was suppressed effectively following the incorporation of single gene resistance to this virus in Australian-bred cultivars. Again, this situation recently changed with a resurgence of JGMV spread being reported in Australian maize and sorghum crops in QLD (see Section 3.3 above). Whether this new development is due to the appearance of a resistance breaking JGMV strain, or the release of new cultivars without JGMV resistance, remains to be investigated (see Section 3.3 above). Similar considerations concerning the appearance of resistance-breaking virus strains also apply to the Australian cereal and oilseed crop breeding efforts currently underway to incorporate single gene resistances to YDV’s in wheat and barley, WSMV in wheat, TuYV in canola, TSV in sunflower and CBTV in cotton (see Section 3 and Section 4 above). Therefore, there is an urgent need not only to monitor both cereal and oilseed crops crops and nearby weeds to search for resistance-breaking virus strains, but also to avoid their introduction by different routes from nearby countries or other world regions (see Section 5.1 above). Such monitoring should be combined with experimental work to understand their biological properties, especially concerning their virulence and propensity for spread locally and over long distances.

### 5.4. Climate Change Threats

The subject of likely climate change induced alterations to epidemics of cereal and oilseed virus diseases around Australia constitutes an issue of considerable magnitude. It can only be dealt with briefly here. A review published in 2009 summarised likely effects on plant virus epidemics, including Australian cereal and oilseed examples, as part of an article also covering virus origins, emergence and evolution [5]. Since then, three global reviews published by Australian researchers have provided more detail on the anticipated effects of increasing levels of greenhouse gasses in the atmosphere and the resulting global warming on plants virus diseases [6,9,11]. Jones and Barbetti [9] used climatic and biological frameworks to establish the probable effects of direct and indirect climate change parameters on the many vector, virus and host parameters that encompass the diversity of plant virus pathosystems. They addressed the multifaceted ramifications of climate change likely to influence plant virus epidemics, and provided comprehensive coverage of the international research then available illustrating the likely effects, including providing cereal and oilseed virus disease predictions for Australia. They predicted that: (i) many critical plant virus epidemic components will be modified in diverse ways, often resulting in epidemic amplification; (ii) important other variables will result from involvement of different kinds of vectors and emergence of formerly unknown viral pathogens; and (iii) increasing difficulties will occur in controlling damaging plant virus epidemics. Jones [6] focused on the likely consequences of climate change influences on plant virus and vector research published between 2011 and 2015 and provided an updated assessment of the likely consequences for plant virus disease epidemics as climate change progresses, including for Australian cereal and oilseed production. More recently, Trębicki [11] focused on changes in plant virus epidemiology likely to occur under future climatic conditions and included examples derived from cereal virus research in south-east Australia. What these reviews tell us about changes relevant to the Australian cereal and oilseed industries is summarised in the rest of this Section.

Although the effects of elevated CO_2_ on insect and mite vectors can include alterations in their population numbers, growth rates, fecundity and feeding, the actual outcome varies greatly between different types of vectors, and even between similar species within the same group of vectors. The same applies to its direct effects on virus multiplication within infected plants, disease severity and plant defense responses to infection as these vary with the virus-host plant pathosystem studied [6,9]. This makes it difficult to predict what changes in virus disease epidemics will arise directly from this parameter (i.e., from elevated CO_2_) in Australia. Hence, the need to focus instead on how virus epidemics are likely to be influenced by the alterations in other climate parameters that result from elevated levels of greenhouse gasses (CO_2_, methane and others) in the atmosphere. These altered climate parameters include rainfall, temperature, wind, and increased climate instability, all of which have major influences on virus epidemic development, and the resulting production losses in cereal and oilseed crops grown in Australia’s diverse agricultural regions.

Where rainfall declines and conditions become warmer, the relative importance of different virus diseases will likely change [5,6,9,39]. For example, WSMV epidemics in wheat crops will likely increase due to more favorable temperature conditions for rapid population build-up of its WCM*a* vector before crops are sown and in the early growing period on volunteer cereal and annual grass hosts. This is because: (i) although there is little WCM population increase under cool conditions, its populations explode under warm conditions, and (ii) it spreads this virus from initial infection foci consisting of infected volunteer or sown wheat plants that grow from WSMV-infected wheat seed (see Section 3.2 above). Therefore, WSMV epidemics do not necessarily require the development of a large living green bridge well before the crop growing period starts. Conversely, the opposite scenario is likely to occur with non-seed borne, persistently transmitted aphid-borne viruses, such as BYDV and CYDV in cereals and TuYV in canola. This is because before widespread and damaging epidemics can develop, both virus and aphid vectors require a substantial living green bridge of weeds and volunteer crop plants to be present between crop growing seasons. In addition, aphids do not require such high temperatures for their populations to increase (see Section 3.1 above). Thus, whereas epidemics of BYDV and CYDV in cereals and TuYV in canola are expected to decline in regions where conditions become hotter and drier outside the growing period, those of WSMV in wheat are likely to be favored by these conditions, and vice versa. The south-west Australian grainbelt provides an example of a major growing region where this scenario is in the process of unfolding [5,6,9,250]. On the one hand, at cooler times of year, cold fronts from the south-west are decreasing in number and penetrating less far inland. This is leading to an expansion of the low rainfall zone associated with contraction of the medium and high rainfall zones and their displacement towards the coast. On the other hand, at warmer times of year, an increase in the number and magnitude of cyclones from the north-west is increasing the rainfall that penetrates inland into the Australian continent, which is reaching the otherwise driest parts of the low rainfall zone most distant from the coast [40,250]. Thus, except in the eastern portion of its low rainfall zone where there is an increase in rainfall outside the growing season, the grainbelt region is drying and this is occurring in conjunction with all-year-round rising temperatures. This scenario is contributing to a decline in the BYDV and CYDV epidemics in cereals that in the past caused substantial losses in the high rainfall zone, the affected region becoming smaller and increasingly limited to cropping areas closer to the coast. It is also creating conditions more favorable for damaging WSMV epidemics to develop in wheat crops in the portion of the low rainfall region most distant from the coast [5,6,9].

Increasing exposure of crops to strong winds arising from cyclones is likely to increase the dispersal of insect and mite vectors in grain growing regions, especially in the northern Australia, thereby dispersing the viruses they carry more widely. However, the heavy rains often associated with them may decrease their vector populations by washing them off plants [9]. Adaptations to crop agronomy designed to conserve and maximize the availability of soil moisture for cropping by minimizing its loss from weed growth and in the soil are increasingly being adopted for Australian cereal production (e.g., minimum tillage, wide row spacing and reduced seeding rate) [42,43,370]. However, they favor the development of aphid-borne virus epidemics because prolonged exposure to bare earth arising from delayed canopy closure attracts insect vector landings in the crop [5,6,9]. In parts of subtropical Australia where increasingly prolonged dry periods between annual cropping seasons occur, this will favor build-up of *B. tabaci* vectors thereby favoring epidemics of viruses they transmit. This is because dry periods when monthly rainfall of less than 80 mm occurs for at least 4 months are required for *B. tabaci* to flourish [9].

Climate instability caused by the climate change process is making it increasingly difficult to achieve effective control of virus diseases of Australian cereal and oilseed crops using integrated disease management approaches (Table 1, Table 2 and Table 3). This applies to control measures needing to be deployed at critical times in order to achieve optimum effectiveness, such as (i) chemical measures that need to coincide with peak insect vector flight times, and (ii) cultural measures designed to avoid exposure of vulnerable young plants, or the maturing crop, to peak insect vector flight times. An example of (i) is applying insecticides, oils or repellents at the most appropriate time, whereas examples of (ii) include manipulation of sowing date and early harvesting of crops when they are likely to provide the greatest benefit. It also applies to other situations where the effectiveness of individual cultural or host resistance control measures is likely to be compromised by a less predictable climate. Examples of this include planting cultivars in situations where unexpectedly high temperatures within the growing season render temperature-sensitive virus resistance genes ineffective, or plants become physiologically less able to withstand virus infections, and planting upwind of infection sources, or behind non-host barrier crops, when traditional prevailing wind patterns change [5,6,9,53]. All of the virus control measures likely to become less effective as climate change proceeds are ones currently available for virus control in Australian cereal and oilseed crops. The ones relied on most often are host resistance genes and insecticide spray applications to kill insect vectors of persistently aphid-borne viruses (see Section 3 and Section 4 above). Examples of current control measures less likely to be rendered ineffective by climate change include: applying herbicides to kill weed and volunteer crop hosts, and sowing healthy seed stocks (phytosanitary); isolation and safe planting distances, minimum tillage and mulches, generating early canopy cover and high plant densities, and fallowing (cultural); applying insecticides as seed dressings or to the soil (chemical); and sowing cultivars with polygenic resistance, or single gene resistance which is not temperature sensitive (host resistance). For each Australian climatic region and cereal or oilseed crop, predictive epidemiological models and decision support systems are required to ensure that cost effective virus disease management is achieved [55]. They are becoming increasingly necessary to ensure that suitable integrated disease management approaches are deployed where and when necessary. However, currently, the only such models available are for YDV’s in cereals and TuYV in canola in south-west Australia [124,235], and these need to be adjusted to take recent alterations in climate into account. Novel approaches to epidemiological modelling may need to be developed as climate becomes less predictable.

Although there are a small number for TuYV in canola, TuMV in mustard and BYDV in wheat, relatively few Australian studies, have examined the likely effects of different climate change parameters on virus diseases of cereal and oilseed crops. The transmission efficiency of TuYV by *M. persicae* is impacted by temperature, with higher infection rates obtained in partially resistant canola cultivars following inoculation at 26 °C than at 16 °C [213], congruous with the optimal temperature range for *M. persicae* population growth and performance [371]. Thus, higher temperatures will likely increase the TuYV transmission by *M. persicae* in partially resistant canola crops. When Australian canola cultivars or breeding lines were inoculated with a TuMV pathotype 8 isolateWA-Ap1, resistance phenotype O proved to be temperature sensitive. This is because, although it developed at 16–18 °C, it was replaced by phenotype R_N_, other resistance phenotypes or segregation for two or more different phenotypes at 25–28 °C [265] (see Section 4.2 above for an explanation of TuMV resistance phenotype codes). Similarly, when this isolate was inoculated to Ethiopian mustard germplasm lines, resistance phenotype O was also temperature sensitive as, although it developed at 16–18 °C, it was often replaced by phenotypes R or R_N_, or by segregation for different phenotypes at 25–28 °C [265]. By contrast, when Indian mustard germplasm lines were inoculated with the same TuMV isolate, although it diminished the delay between TuMV inoculation and symptom development, changing temperature from 16 °C to 28 °C made no difference to resistance phenotype [208]. When wheat plants infected with BYDV-PAV were held at 5–16 °C or 10–21 °C day-night temperatures, its titer increased, and its symptoms developed sooner under the higher temperature regime [372]. Also, when wheat plants infected with BYDV-PAV were held under ambient (= 400 μmol mol^−1^) or elevated (650 μmol mol^−1^) CO_2,_ its titer was increased by elevated CO_2_ [373]. In addition, when healthy or BYDV-PAV infected wheat plants infested with BYDV’s *R. padi* vector were held under ambient CO_2_ (385 μmol mol^−1^) or elevated CO_2_ (650 μmol mol^−1^), on healthy plants elevated CO_2_ caused the aphid vector population to diminish and its feeding damage to increase, whereas in plants infected with BYDV-PAV neither parameter was altered [374]. A further study found the incidence of BYDV increased in wheat plots exposed to elevated CO_2_ (550 μmol mol^−1^) in an Australian free-air CO_2_ enrichment facility [375].

To prepare for future climate change induced epidemics of virus diseases in cereal and oilseed crops in Australia, a much greater emphasis on research on this subject is required. Such research would need to focus on the effects arising from increased greenhouse gasses, temperatures or wind speeds, insufficient or excessive rainfall, and extreme weather events upon epidemics of Australia’s major current virus disease threats to cereal and oilseed production (YDVs and WSMV in cereals, and TuYV, TuMV, TSV and CBTV in oilseeds (see Section 3 and Section 4 above). It would also need to: (i) address the increasing difficulties in controlling epidemics of these virus diseases arising from climate instability and unpredictability (see previous paragraphs in this Section); and (ii) provide the epidemiological information needed to prepare for future outbreaks of damaging virus diseases likely to become established in the future in different regions due to the changing climate (see Section 5.1 above). In 2012, Jones and Barbetti [9] provided a detailed list of 10 priorities for future climate change research on virus diseases in global agriculture, most of which are relevant to virus diseases damaging Australian crops. Since, then initial steps have been made in investigating the impacts of elevated temperature and CO_2_ on virus diseases of cereals and oilseeds in Australia (see previous paragraphs in this Section), but a great deal more needs to be done before the country is ready to face what is likely to occur in the future. More climate change scenario modelling is needed to establish when significant viral pathogens or vectors are likely to increase causing damaging virus epidemics in crops or invade regions where conditions were formerly unsuitable for them. Moreover, initial experiments studying the effects of diverse climate change parameters on viral diseases under controlled environment conditions need to be followed up by examining their effects on virus epidemics occurring in field situations, including in FACE facilities, and in crops growing in regions with high temperatures or where flood or drought conditions occur.

### 5.5. Insufficient Industry Awareness

Although in the past the importance of crop virus diseases was better understood by Australian cereal and oilseed industries, more recently this knowledge has declined. To comprehend this situation, it is important to understand long term trends that have been influencing the overall Australian broadacre grain industry since at least the 1950’s associated with climate change, adoption of new technologies and globalization of agriculture (see Section 2.1 above). In particular, there is a continuing increase in the scale and efficiency of individual broadacre farms, associated with a decline in rural populations. These trends are accompanied by a decline in the number of active grains industry researchers (particularly those dedicated to applied virus disease research), and of regional research extension staff belonging to state government and other institutions [376,377,378]. These trends are exacerbating the underestimation of virus diseases due to characteristics inherent to virus symptom recognition in crop foliage, seasonal variation in epidemic occurrence and control requirements. Firstly, in any given growing season or grain growing region, there are diverse viruses that cause disease in different crops, and, being greatly influenced by climatic conditions, their outbreaks are often sporadic. Their sporadic occurrence means that consultants and farmers may not be exposed to virus disease symptoms as often as they are to other crop disease symptoms, such as those caused by some common fungal pathogens. Furthermore, common virus diseases sometimes cause subtle symptoms easily missed by the untrained eye, and the more obvious symptoms that occur with virus diseases are often confused with nutrient deficiency, herbicide damage or abiotic stress factors, e.g., [56,59,73,379]. As most farmers, consultants and regional researchers now have far larger cropping areas to cover when monitoring for crop diseases than they did in the past, all this means that virus diseases are more likely to be overlooked or misdiagnosed. Unfortunately, this has resulted in a significant underestimation of virus disease incidence and impact over time across Australian grain production regions. As the major funding bodies for virus disease research in Australia are paid by grower levies and so are responsive to farmer views over research priorities, this underestimation has, in turn, resulted in a corresponding underassessment of the need for virus disease research by the funding bodies themselves. Thus, adequate funding for research with outcomes that improve virus disease understanding and ensure their effective management is often lacking and at risk of yet further depreciation. Furthermore, this situation has contributed to cases in which Australian grains industry research providers have allocated funds for research on virus diseases of cereal, oilseed and other crops to non-virologists (e.g., entomologists, fungal plant pathologists or molecular biologists), instead of to dedicated plant virologists with practical experience of the epidemiology and control of virus diseases in the field. This has further contributed to a failure to address practical virus disease issues adequately. Lastly, virus diseases cannot be controlled curatively (i.e., with foliar sprays) in contrast to common fungal pathogens that consultants and farmers are accustomed to deal with. Moreover, particularly for farmers operating on large farms across Australian grainbelts, some components of the disease management strategies, especially phytosanitary and cultural control measures, required to reduce spread of virus diseases may conflict with agronomic, time and scale constraints associated with mainstream grains industry ‘best practice’. If researchers and industry fail to collaborate to alleviate these serious issues, Australian cereal and oilseed industries will continue to experience poorly managed virus disease epidemics, and the consequent losses in profits associated with lower yields and associated produce quality.

To address these problems, a vigorous and sustained industrywide extension effort focused on cereal and oilseed virus disease identification, epidemiology, impact and integrated disease management is required. Such an extension effort requires delivery in simple readily understood language. Australian federal and state government agriculture departments and their crop virus disease experts need to provide carefully targeted virus identification and management courses and extend this information widely via media platforms such as rural radio, farmer magazines, social media and the rural press. Furthermore, extension information generated by surveillance programs and risk forecasting needs to be extended in a targeted and timely manner on these platforms. In addition, virus disease researchers need to engage with, and collaborate frequently with, farmers and consultants, including doing fully replicated, large-scale field experiments, which are demonstrated to farmers at field days, on rural research stations, and conducting joint experimental trials and demonstrations with local farmer groups and consultancy agencies. A challenge for future virus disease research in Australian broadacre crops will be to ensure the development of virus disease management strategies that are compatible with the current paradigm of production, i.e., the need to be time efficient and operate at scale. At present, the best way forward is to combine available phytosanitary, chemical and cultural measures with provision of high yielding virus resistant commercial cereal and oilseed cultivars. Currently, large swathes of susceptible cereal and oilseed cultivars arebeing produced as monocultures in Australia, increasing the risk of significant virus disease epidemics over vast areas of cropping. Due to the very large scale of production, the use of drones, remote imaging, ‘smart’ insect vector trapping and forecasting models combined with decision support systems are beginning to playing important roles in the Australian grains industry and should be further explored for their utility for virus disease management [55,61,124,211,235,380]. In terms of research funding, ideally virus disease research projects should be led by a plant virologist with practical experience of virus epidemiology and control in the field, but also be associated as part of the overall research team with collaborators having other relevant skills, such as vector entomologists, agronomists, statisticians, modelers, molecular biologists and experienced extension staff.

## 6. Conclusions

Here we describe the considerable research achievements made over the last 70 years in providing a sound understanding of the biology and epidemiology of the virus diseases of cereal and oilseed crops of greatest economic importance to Australia, and in developing effective management tactics that control them. In addition, we highlight the growing threat to effective management of cereal and oilseed virus diseases arising from: (i) increasing difficulties in controlling them due to the escalating levels of climate instability and extreme weather events; (ii) the rise in insecticide resistance in insect vectors, combined with the likelihood of a future ban on several insecticides currently relied upon to manage them; (iii) the development of virus strains that break resistance genes being used in breeding new virus-resistant cultivars; and (iv) the lack of sufficient industry awareness about the importance of virus diseases. We also provide detailed recommendations for future research needed to address these threats and emphasize the major biosecurity risk to Australian cereal and oilseed industries posed by damaging viruses and their virus vector species spreading from other countries.

With the sole exception of rice and linseed, virus diseases are known to occur in all of the diverse range of 19 different cereal and oilseed crops currently grown in the Australian continent’s temperate, Mediterranean, subtropical and tropical cropping regions. The cereal virus diseases currently of greatest cause for concern are those caused by BYDV and CYDV in wheat, barley and oats, WSMV in wheat, and JGMV in maize, sweet corn and sorghum. Those of greatest concern for oilseeds are TuYV and TuMV in canola and Indian mustard, TSV in sunflower and CBTV in cotton. In the past, two of these viruses (JGMV in maize, sweet corn and sorghum, and TuMV in canola) have been controlled effectively by planting crop cultivars with virus resistance genes, but this situation is now changing due to the appearance of resistance-breaking virus strains or planting new cultivars lacking virus resistance. In addition, there are 23 other viruses infecting one or more crops that are currently of less importance, nine of which infect nine different cereal crops and 14 of which infect eight different oilseed crops. However, several of these have the potential to cause greater losses in the future. The cereal viruses of greatest concern should they become established in Australia include MDMV, MSMV, MRDV and the virus complex causing MLND in maize and sweet corn crops, and RHBV, RYMV and the virus complex causing RTD in rice. In cooler, wetter regions of Australia, several soil-borne viruses transmitted by *P. graminis* pose a threat to wheat and/or barley crops (BaMMV, BaYMV, CWMV, SBCMV, SBWMV, WSSV, WYMV), and oats (OGMV, OGSV), and in subtropical or tropical regions to maize, sweet corn, sorghum, pearl millet and/or rice crops (PCV, IPCV). The oilseed viruses of greatest concern include CLRDV, CLCrDV and CLCuVs in cotton; and GBNV, PCV and the virus complex causing the GRD in peanut and/or soybean. However, currently, there do not appear to be any obvious external biosecurity threats to Australian canola and Indian mustard crops from virus diseases present elsewhere.

Despite the considerable knowledge accumulated in the past, recent information available from (i) virus disease surveys, epidemiology research and yield loss studies from across Australia, and (ii) increased understanding of the growing threats to their effective management from climate instability and extreme weather events, insecticide resistance breakdown, and virus resistance breaking strains, reveal that cereal and oilseed virus diseases and their management require a considerable further research effort. Greater emphasis on traditional research involving field experiments and studies on plant virus disease epidemiology and management is required. Also, a vigorous extension effort is needed to ensure farmers understand the threat virus diseases pose to their crops. Moreover, there is a pressing need to understand how rapidly changing farming systems and alterations in global market requirements, are likely to alter future virus disease epidemics, and the extent to which this will require modification of disease management approaches for cereal and oilseed crops under changing circumstances. Such knowledge will enable farmers to maximize their profits in years when yields are high and minimize their losses in years when they are low. In addition, the climate in Australia’s cereal and oilseed growing regions is changing rapidly as maximum temperatures rise, and rainfall within, or between, growing seasons declines or increases, and becomes more erratic. This is causing rising uncertainty over when virus control measures are needed, and when to intervene within growing crops. This uncertainty will increasingly compromise decision making over which control measures to use in order to optimize the desired control outcome. In addition, farmers need reliable early warning systems based on vector and virus loads from both the current and previous growing season. Such early warning systems enable estimates of risk, inform in-crop monitoring decisions, and assist with the timely and targeted implementation of control strategies. Furthermore, the current approach of utilizing historical data and new research approaches to optimize disease modelling and decision support systems is important. The principal focus of future research on the better understood virus diseases of cereals and oilseeds should involve ensuring that epidemiological models and decision support systems already available (BYDV in wheat, TuYV in canola) are deployed nationally. Additional local research on their epidemiology and management that complements any research findings already available may be required leading to the development of epidemiological models and decision support systems suitable for use in different parts of the continent.

Given the magnitude of the threat posed to Australian cereal and oilseed crops should damaging virus diseases, or any of their insect, mite or protist vectors, that occur in one or more other world regions become established in the country, stringent biosecurity measures are required in pre-border, border and post-border situations (see Section 5.1 above). Although much has already been achieved, and Australian plant biosecurity measures are among the strictest in the world, the country has a unique advantage in still being free of many damaging virus diseases. In order to help ensure this advantage is maintained, more research is needed to strengthen the procedures currently used and incorprate the latest advances in technology that are capable of further improving them.

Finally, preparation of a revised version the book ‘Viruses of Plants in Australia’ that brings it up-to-date by summarizing all new plant virus identifications, and recent studies on the biology, epidemiology and management of each virus, and by confirming, or otherwise, previous assumptions about virus identifications in the light of recent molecular studies, would provide an extremely valuable resource for Australia.

## Figures and Tables

**Table 1:** The selectivity and activity against primary virus source(s) and virus spread exhibited by different control measures.

Method	Measure	Selectivity		Primary Source (X_o_)		Rate of Spread (r)	
		Low	High	External	Internal	Early	Late
Phytosanitary, against virus	General hygiene	+	−	+	+	+	−
	Roguing	+	+	−	+	+	−
	Healthy seed, seed sieving, seed therapy	+	−	−	+	+	−
Phytosanitary, against vector and virus	Weed control, soil solarization, fallowing	+	−	−	+	+	−
Cultural	Isolation, safe planting distances	+	−	+	−	+	−
	Planting upwind, non-host barrier, large field size, windbreaks	+	−	+	−	+	−
	Manipulate sowing date to avoid peak vector numbers	+	−	−	−	+	−
	Mini7mum tillage, groundcover, mulches, reflective surfaces	+	−	−	−	+	−
	Early canopy cover, high plant density, narrow row spacing	+	−	+	+	−	+
	Early harvest, early maturing cultivar	+	−	−	−	−	+
	Crop and weed free periods, single and phased rotations	+	−	+	+	+	−
Chemical, against vector	Specific, regular foliar applications (PTO)	−	+	−	−	+	+
	General, regular foliar applications (PTO)	+	−	−	−	+	+
	Specific, seed dressings (PTO)	−	+	−	−	+	−
	General, seed dressings (PTO)	+	−	−	−	+	−
	Specific, in furrow or soil before or directly after planting (PTO)	−	+	−	−	+	−
	General, in furrow or soil before or directly after planting (PTO)	+	−	−	−	+	−
Natural host resistance, against virus	Partial, polygenic	−	+	−	−	+	(+)
	Strain-specific, single gene, resistance-breaking strain absent	−	+	−	−	+	+
	Extreme resistance, single gene	−	+	−	−	+	+
Natural host resistance, against vector	Partial, polygenic	−	+	−	−	+	(+)
	Specific, single gene, resistance-breaking vector race absent	−	+	−	−	+	(+)

+ = active; − = inactive; (+) = partially active; PTO = persistent insect transmission only with arthropod vectors (ineffective with non-persistent transmission).

**Table 2:** The Most important viruses that infect cereal and oilseed crops in Australia.

Crop Type	Virus	Virus Genus	Crops Affected	Main Foliage Symptoms	Vector	Seed Transmission	Maximum % Yield Loss Recorded	Seed Quality Defect	Region Recorded in ^a^
Cereal	Barley yellow dwarf virus (BYDV)	*Luteovirus*	Barley, wheat, oats, triticale, rye	Chlorosis, reddening, stunting	Aphids	No	80%	Shriveled grain, reduced seed size, weight and protein content	STE, MED, TE
	Cereal yellow dwarf virus (CYDV)	*Polerovirus*	Barley, wheat, oats, triticale, rye	Chlorosis, reddening, stunting	Aphids	No	No data	No data	STE, MED, TE
	Johnsongrass mosaic virus (JGMV)	*Potyvirus*	Maize, sweet corn, sorghum, pearl millet	Mosaic, reddening, necrosis, stunting	Aphids	Yes	92%	Reduced size	TN, STE
	Wheat streak mosaic virus (WSMV)	*Tritimovirus*	Wheat	Mosaic, chlorosis, streaking, stunting	Mites	Yes	83%	Shriveled grain	STE, MED, TE
Oilseed	Cotton bunchy top virus (CBTV)	*Polerovirus*	Cottonseed	Mosaic, deformation, stunting	Aphids	No	100%	No data	TN, STE
	Tobacco streak virus (TSV)	*Ilarvirus*	Peanut, safflower, soybean, sunflower	Chlorosis, deformation, necrosis, stunting, plant death	Thrips	No	70%	Reduced size	TN, STE, TE
	Turnip mosaic virus (TuMV)	*Potyvirus*	Canola, Indian mustard	Mosaic, stunting	Aphids	No	84%	Reduced size	STE, MED, TE
	Turnip yellows virus (TuYV)	*Polerovirus*	Canola, Indian mustard	Chlorosis, reddening, stunting	Aphids	No	46%	Fewer seeds, reduced oil content, increased erucic acid, glucosinolates	STE, MED, TE

Information sources: Buchen-Osmond (1988) [13], CABI Data Sheets, AAB/CMI Descriptions of Plant Viruses, VIDE Data Base, Searches using Google and Google Scholar. ^a^ Australian grain growing regions: tropical north (TN), subtropical east (STE), Mediterranean (MED), and temperate (TE).

**Table 3:** Other viruses that infect cereal and oilseed crops in Australia.

Crop Type	Virus	Virus Genus	Crops Affected	Main Foliage Symptoms	Vector	Seed Transmission	Maximum % Seed Yield Loss Recorded	Seed Quality Defect	Region Recorded in ^a^
Cereal	Barley stripe mosaic virus	*Hordeivrus*	Barley, wheat, rye	Stripey mosaic, sometimes necrotic	Contact only	Yes	35%	Reduced size	STE, MED, TE
	Barley virus G	*Polerovirus*	Barley, pearl millet	Mosaic, yellow striping, necrosis	Aphid	No	No data	No data	TE
	Barley yellow striate mosaic virus (Synonym: Cereal striate mosaic virus, maize sterile stunt virus)	*Nucleorhabdovirus*	Barley, maize, wheat, oats, rye	Chlorotic stipes, mosaic, reddening, stunting, sterility	Planthopper	No	No data	No data	TE, STE
	Cereal chlorotic mottle virus	*Nucleorhabdovirus*	Barley, maize, wheat, oats, rye	Chlorotic stipes, stunting and sterility	Leafhopper	No	No data	No data	TE, STE
	Chloris striate mosaic virus (Synonym: Wheat striate mosaic)	*Mastrevirus*	Barley, oats, maize, wheat	Chlorotic steaking, striate mosaic, stunting	Leafhopper	No	No data	No data	TE, STE
	High plains wheat mosaic virus (HPWMoV)	*Emravairus*	Wheat, maize	Mosaic, chlorosis, streaking, stunting	Mites	Yes	No data,	No data,	STE, MED
	Maize stripe virus	*Tenuivirus*	Maize, sweet corn, sorghum	Chlorotic stripes, apical bending, stunting, premature death	Planthopper	No	76%	No data	TN, STE, MED, TE
	Maize yellow dwarf virus (MYDV)	*Polerovirus*	Maize, sweet corn, barley, oats, wheat	Chlorosis, reddening, stunting	Aphids	No	No data,	No data,	STE, MED, TE
	Sugarcane mosaic virus (SMV)	*Potyvirus*	Maize, sorghum, pearl millet	Mosaic, ringspots, necrosis	Aphid	No	N/A	N/A	TN, STE
Oilseed	Alfalfa mosaic virus (AMV)	*Alfamovirus*	Soybean	Mosaic, yellow spotting, stunting	Aphid	Yes	48%	Fewer mottled seeds	STE, MED, TE
	Broccoli necrotic yellows virus (BNYV)	*Cytorhabdovirus*	Canola	Asymptomatic	Aphid	No	No data	No data	STE, TE
	Capsicum chlorosis virus (CaCV)	*Orthotospovirus*	Peanut	Mottle, necrotic spotting, reduced internode length, terminal growing points wilt and die	Thrips	No	No data	No data	TN, STE
	Cauliflower mosaic virus (CaMV)	*Caulimovirus*	Canola, Indian mustard	Vein clearing, vein banding, chlorotic rings, mosaic	Aphids	No	90%	Reduced size	STE, MED, TE
	Cowpea mild mottle virus (CpMMV)	*Calarvirus*	Soybean	Mosaic, leaf distortion, stunting, pod distortion, fewer pods	Whitefly	Yes	16%	Reduced size	STE
	Cucumber mosaic virus (CMV)	*Cucumovirus*	Sunflower	Mosaic, chlorotic rings, brown streaking of petioles and stems, stunting	Aphid	No data	No data	Shriveled seed	STE
	Glycine mosaic virus (GlMV)	*Comovirus*	Soybean	Mosaic	No data	No data	No data	No data	STE
	Lucerne Australian latent virus (LALV)	*Nepovirus*	Soybean	No data	No data	Yes	No data	No data	STE, TE
	Passionfruit woodiness virus (PWV)	*Potyvirus*	Peanut, soybean	Mosaic, ringspots, leaf deformation	Aphid	No	No data	No data	STE, MED, TN
	Peanut mottle virus (PMoV)	*Potyvirus*	Peanut, soybean	Mottle, leaf distortion, mild stunting	Aphid	Yes	40%	Reduced size, malformation, discoloration	STE
	Pelargonium zonate spot virus (PZSV)	*Anulavirus*	Sunflower	Bright chlorotic rings	None	Yes (only in tomato)	No data	No data	STE, MED
	Soybean mosaic virus (SoMV)	*Potyvirus*	Soybean	Mosaic, deformation, stunting	Aphid	Yes	35% (none reported in Australia)	Reduced size, seed coat mottling.	STE, MED, TE
	Tomato spotted wilt virus (TSWV)	*Orthotospovirus*	Peanut	Mosaics, chlorotic or necrotic streaking or spotting, ringspots, line patterns, general necrosis, wilting, stunting	Thrips	No	100%	Reduced size, cracking of seedcoat	STE, MED, TE, TN
	Watermelon mosaic virus (WMV)	*Potyvirus*	Sesame	N/A	Aphid	No	No data	No data	STE

Information sources: Buchen-Osmond et al. [13], CABI Data Sheets, AAB/CMI Descriptions of Plant Viruses, VIDE Data Base, Searches using Google and Google Scholar. ^a^ Australian grain growing regions: tropical north (TN), subtropical east (STE), Mediterranean (MED), and temperate (TE). N/A = Not available.

**Table 4:** Cereal and oilseed virus diseases that pose a biosecurity threat to Australia.

Syndrome	Virus or Virus Complex	Virus Genus	Cereal and Oilseed Crops Affected	Vector	Current Distribution	Disease Impact
Barley mild mosaic disease	Barley mild mosaic virus (BaMMV)	*Bymovirus*	Barley	Plasmodiophorid protist (*Polymyxa graminis*)	Europe, East Asia	Major yield losses in some years
Barley yellow mosaic disease	Barley yellow mosaic virus (BaYMV)	*Bymovirus*	Barley	Plasmodiophorid protist (*Polymyxa graminis*)	Europe, Middle East, East Asia, North America	Major yield losses in some years
Chinese wheat mosaic disease	Chinese wheat mosaic virus (CWMV)	*Furovirus*	Wheat	Plasmodiophorid protist (*Polymyxa graminis*)	East Asia	Major yield losses in some years
Cotton blue disease	Cotton leafroll dwarf virus (CLRDV)	*Polerovirus*	Cotton	Aphid (*Aphis gossypii*)	Sub-Saharan Africa, Middle East, Southeast Asia, North and South America	Devastating yield losses; major deterrent to cotton cultivation. Likely losses in grain legumes.
Cotton leaf curl disease complex	Cotton leaf curl viruses (CLCuVs)	*Begomovirus(es*)	Cotton	Whitely (*Bemisia tabaci*)	Indian subcontinent, East Asia, Southeast Asia, North, East and West Africa	Devastating yield losses; crop failure; major deterrent to cotton cultivation
Cotton leaf crumple disease	Cotton leaf crumple virus (CLCrV)	*Begomovirus*	Cotton	Whitely (*Bemisia tabaci*)	Indian subcontinent, Central and North America	Major yield losses in epidemic years
Groundnut bud necrosis disease	Groundnut bud necrosis virus (GBNV)	*Orthotospovirus*	Peanut, soybean	Thrips (*Thrips palmi* and *Frankliniella shultzei*)	Indian subcontinent, Central and East Asia, Southeast Asia	Severe yield losses with early infection
Groundnut rosette tri-partite disease complex (GRD)	Groundnut rosette virus (GRV), groundnut rosette assistor virus (GRAV), and virus satellite	*Umbravirus* (GRV), *Luteovirus* (GRAV), virus satellite tri-partite complex	Peanut	Aphid (*Aphis craccivora*)	Sub-Saharan Africa and offshore islands, including Madagascar	Devastating yield losses in some years, crop failure; major deterrent to peanut cultivation
Peanut clump disease	Peanut clump virus (PCV), Indian peanut clump virus (IPCV)	*Pecluvirus*	Barley, maize, sweet corn, peanut, pearl millet, sorghum, rice, wheat	Plasmodiophorid protist (*Polymyxa graminis*)	Northern region of Sub-Saharan Africa (PCV), Indian subcontinent (IPCV)	Major yield losses in epidemic years
Maize dwarf mosaic disease	Maize dwarf mosaic virus (MDMV)	*Potyvirus*	Maize, sweet corn, sorghum, oats	Many aphid species (including *Rhopalosiphum maidis, Myzus persicae, Aphis craccivora, Aphis gossypii, Brevicoryne brassicae* and *Rhopalosiphum padi*.	Europe, North, Central and South America, North and Sub-Saharan Africa, Middle Est, Central and East Asia, Indian Subcontinent, Southeast Asia	Most important and damaging virus disease of sorghum worldwide. Severe losses in susceptible maize hybrids, and sweet corn
Maize lethal necrosis bi-partite disease complex (MLND)	Maize chlorotic mottle virus (MCMV), plus sugarcane mosaic virus (SCMV), maize dwarf mosaic virus (MDMV) or wheat streak mosaic virus (WSMV)	*Machlomovirus* (MCMV), plus *Potyvirus* (SCMV, MDMV) or *Tritimovirus* (WSMV)	Maize, sweet corn (MLND). Barley, maize, sweet corn, sorghum, pearl millet, wheat (MCMV). Several cereal crops (MDMV, SCMV, WSMV)	Beetle and thrips species (including the thrips *Frankliniella williamsi*) (MCMV). Several aphid species (SCMV, MDMV). Eriophyid mite (*Aceria tosichella*) (WSMV)	East and Central Africa, East Asia, Southeast Asia, North and South America, Europe	Widespread devastating yield losses, crop failure, food shortages, major deterrent to maize and sweet corn cultivation
Maize rough dwarf disease	Maize rough dwarf virus (MRDV)	*Fijivirus*	Maize, sweet corn	*Planthopper (Laodelphax striatellus*)	Mediterranean region and Middle East	Devastating yield losses in hybrid maize cultivars; major threat to maize and sweet corn cultivation
Maize streak disease	Maize streak mosaic virus (MSMV)	*Mastrevirus*	Maize, sweet corn	Leafhoppers (*Cicadulina mbila,* nine other *Cicadulina* species)	Sub-Saharan Africa	Widespread devastating yield losses in some years, crop failure; major deterrent to maize and sweet corn cultivation
Oat mosaic disease	Oat mosaic virus (OMV)	*Bymovirus*	Oats	Plasmodiophorid protist (*Polymyxa graminis*)	Europe, North America	Major yield losses in some years
Oat golden stripe disease	Oat golden stripe virus (OGSV)	*Furovirus*	Oats	Plasmodiophorid protist (*Polymyxa graminis*)	Europe	Major yield losses in some years
Rice hoja blanca disease	Rice hoja blanca virus (RHBV)	*Tenuivirus*	Rice	Planthopper (*Sogatodes orizicola*)	South, Central and North America	Devastating yield losses in some years, crop failure
Rice tungro bi-partite disease complex	Rice tungro bacilliform virus (RTBV),plus rice tungro spherical virus (RTSV)	*Tungrovirus* (RTBV), *Waikavirus* (RTSV)	Rice	Several leafhopper vector species transmit both viruses, *Nephotettix virescens* most efficient vector	Southeast Asia, East Asia (China),the Indian subcontinent	Widespread devastating yield losses, famine. Major deterrent to rice cultivation
Rice yellow mottle disease	Rice yellow mottle virus (RYMV)	*Sobemovirus*	Rice	Contact, beetles, mammals soil and water	East, Central and West Africa, Madagascar	Widespread devastating yield losses; major deterrent to rice cultivation
Soil-borne cereal mosaic disease	Soil-borne cereal mosaic virus (SBCMV)	*Furovirus*	Wheat, rye, triticale	Plasmodiophorid protist *Polymyxa graminis*	Europe	Major yield losses in some years
Soil-borne wheat mosaic disease	Soil-borne wheat mosaic virus (SBWMV)	*Furovirus*	Wheat, barley, rye, triticale	Plasmodiophorid protist *Polymyxa graminis*	Europe, New Zealand, Asia, South America, North America	Major yield losses in some years
Wheat yellow mosaic disease	Wheat yellow mosaic virus (WYMV)	*Bymovirus*	Wheat	*Plasmodiophorid protist (Polymyxa graminis*)	Asia	Major yield losses in some years
Wheat spindle streak mosaic disease	Wheat spindle streak mosaic virus (WSSMV)	*Bymovirus*	Wheat, rye, triticale	Plasmodiophorid protist (*Polymyxa graminis*)	North America, Europe	Major yield losses in some years
Triticum mosaic disease	Triticum mosaic virus (TriMV)	*Poacevirus*	Barley, wheat	Eryophid mite (*Aceria tosichella*)	North America	Major losses in mixed infection due to synergistic interaction with wheat streak mosaic virus

Information sources: CABI Data Sheets, AAB/CMI Descriptions of Plant Viruses, VIDE Data Base, Searches using Google and Google Scholar.

## Data Availability

Not applicable.
